# Colonization factors among enterotoxigenic *Escherichia coli* isolates from children with moderate-to-severe diarrhea and from matched controls in the Global Enteric Multicenter Study (GEMS)

**DOI:** 10.1371/journal.pntd.0007037

**Published:** 2019-01-04

**Authors:** Roberto M. Vidal, Khitam Muhsen, Sharon M. Tennant, Ann-Mari Svennerholm, Samba O. Sow, Dipika Sur, Anita K. M. Zaidi, Abu S. G. Faruque, Debasish Saha, Richard Adegbola, M. Jahangir Hossain, Pedro L. Alonso, Robert F. Breiman, Quique Bassat, Boubou Tamboura, Doh Sanogo, Uma Onwuchekwa, Byomkesh Manna, Thandavarayan Ramamurthy, Suman Kanungo, Shahnawaz Ahmed, Shahida Qureshi, Farheen Quadri, Anowar Hossain, Sumon K. Das, Martin Antonio, Inacio Mandomando, Tacilta Nhampossa, Sozinho Acácio, Richard Omore, John B. Ochieng, Joseph O. Oundo, Eric D. Mintz, Ciara E. O’Reilly, Lynette Y. Berkeley, Sofie Livio, Sandra Panchalingam, Dilruba Nasrin, Tamer H. Farag, Yukun Wu, Halvor Sommerfelt, Roy M. Robins-Browne, Felipe Del Canto, Tracy H. Hazen, David A. Rasko, Karen L. Kotloff, James P. Nataro, Myron M. Levine

**Affiliations:** 1 Programa de Microbiología y Micología, Instituto de Ciencias Biomédicas, Facultad de Medicina, Universidad de Chile, Santiago, Chile; 2 Center for Vaccine Development, University of Maryland School of Medicine, Baltimore, MD, United States of America; 3 Department of Microbiology and Immunology at Institute of Biomedicine, University of Göteborg, Göteborg, Sweden; 4 Centre pour le Développement des Vaccins du Mali (CVD-Mali), Bamako, Mali; 5 National Institute of Cholera and Enteric Diseases, Kolkata, India; 6 Department of Paediatrics and Child Health, the Aga Khan University, Karachi, Pakistan; 7 International Centre for Diarrhoeal Disease Research, Mohakhali, Dhaka, Bangladesh; 8 Medical Research Council (United Kingdom) Unit, Fajara, The Gambia; 9 Centro de Investigação em Saúde da Manhiça, Maputo, Mozambique; 10 ISGlobal, Barcelona Ctr. Int. Health Res. Hospital Clínic—Universitat de Barcelona, Barcelona, Spain; 11 Kenya Medical Research Institute/Centers for Disease Control and Prevention, Kisumu, Kenya; 12 Global Disease Detection Division, Kenya Office of the US Centers for Disease Control and Prevention, Nairobi, Kenya; 13 Division of Foodborne, Waterborne and Environmental Diseases, Centers for Disease Control and Prevention, Atlanta, Georgia, United States of America; 14 Centre of Intervention Science in Maternal and Child Health, Centre for International Health, Department of Global Public Health and Primary Care, University of Bergen, Bergen, Norway; 15 Norwegian Institute of Public Health, Oslo, Norway; 16 Department of Microbiology and Immunology, Peter Doherty Institute for Infection and Immunity, The University of Melbourne, Victoria, Australia; 17 The Institute of Genomic Sciences, University of Maryland School of Medicine, Baltimore, Maryland, United States of America; University of Texas Medical Branch, UNITED STATES

## Abstract

**Background:**

Enterotoxigenic *Escherichia coli* (ETEC) encoding heat-stable enterotoxin (ST) alone or with heat-labile enterotoxin (LT) cause moderate-to-severe diarrhea (MSD) in developing country children. The Global Enteric Multicenter Study (GEMS) identified ETEC encoding ST among the top four enteropathogens. Since the GEMS objective was to provide evidence to guide development and implementation of enteric vaccines and other interventions to diminish diarrheal disease morbidity and mortality, we examined colonization factor (CF) prevalence among ETEC isolates from children age <5 years with MSD and from matched controls in four African and three Asian sites. We also assessed strength of association of specific CFs with MSD.

**Methodology/Principal findings:**

MSD cases enrolled at healthcare facilities over three years and matched controls were tested in a standardized manner for many enteropathogens. To identify ETEC, three *E*. *coli* colonies per child were tested by polymerase chain reaction (PCR) to detect genes encoding LT, ST; confirmed ETEC were examined by PCR for major CFs (Colonization Factor Antigen I [CFA/I] or Coli Surface [CS] antigens CS1-CS6) and minor CFs (CS7, CS12, CS13, CS14, CS17, CS18, CS19, CS20, CS21, CS30). ETEC from 806 cases had a single toxin/CF profile in three tested strains per child. Major CFs, components of multiple ETEC vaccine candidates, were detected in 66.0% of LT/ST and ST-only cases and were associated with MSD versus matched controls by conditional logistic regression (p≤0.006); major CFs detected in only 25.0% of LT-only cases weren’t associated with MSD. ETEC encoding exclusively CS14, identified among 19.9% of 291 ST-only and 1.5% of 259 LT/ST strains, were associated with MSD (p = 0.0011). No other minor CF exhibited prevalence ≥5% and significant association with MSD.

**Conclusions/Significance:**

Major CF-based efficacious ETEC vaccines could potentially prevent up to 66% of pediatric MSD cases due to ST-encoding ETEC in developing countries; adding CS14 extends coverage to ~77%.

## Author summary

Enterotoxigenic *Escherichia coli* (“ETEC”) were found to be one of the four most consistently important agents that cause moderate-to-severe diarrhea among children <5 years of age in a large case-control study, the Global Enteric Multicenter Study, performed in four countries in sub-Saharan Africa and three in South Asia. ETEC attach to the lining of the human small intestine by means of protein colonization factors (CFs), after which bacterial toxins stimulate intestinal secretion resulting in diarrhea. Moderate-to-severe diarrhea in young children in developing countries can lead to malnutrition and death. Vaccines are being developed to prevent ETEC diarrhea and its consequences. Several ETEC vaccines aim to stimulate antibodies (protective proteins) that will bind CFs and prevent the bacteria from attaching to intestinal cells, which should, in turn, prevent ETEC diarrhea. Different types of CFs exist. To guide the development of vaccines intending to provide broad protection against ETEC, one must know the frequency with which the different major CFs are produced by ETEC. This paper reports an extensive systematic survey of ETEC CFs and provides helpful information to guide the development of ETEC vaccines.

## Introduction

Enterotoxigenic *Escherichia coli* (ETEC) cause diarrheal disease in children <5 years of age in developing countries and travelers’ diarrhea among persons from industrialized countries who visit developing countries [1,2]. Human ETEC strains can produce a heat-labile enterotoxin (LT) that resembles cholera toxin and one or more heat-stable enterotoxins (ST) including human ST (STh) or porcine ST (STp). Strains can produce both LT and ST (LT/ST) or be ST-only or LT-only. Most ETEC encode colonization factors (CFs) that allow the pathogen to attach to proximal small intestine enterocytes, the critical site of host-parasite interaction, before expressing enterotoxins that decrease villus tip cell absorption and evoke secretion of electrolytes and water by crypt cells [3].

Three main families of Colonization Factor Antigens (CFAs) are encoded by ETEC that cause diarrhea in humans including CFA/I, CFA/II and CFA/IV [3]. CFA/I is the sole member of the first family. CFA/II strains encode coli surface (CS) antigen 3 (CS3) alone or in combination with CS1 or CS2 [3], while CFA/IV strains encode CS6 alone or in conjunction with CS4 or CS5 [3]. CFA/I, CS1, CS2, CS4 and CS5 are rigid fimbriae ~6–7 nm in diameter, CS3 are thin flexible fibrillae 2–3 nm in diameter [4], and CS6 morphology is nondescript.

ETEC vaccines intending to stimulate anti-CF immunity, with or without accompanying antitoxin immunity, are in clinical development. These include purified fimbriae or tip adhesins [5], inactivated fimbriated ETEC [6], attenuated ETEC expressing CFs [7], bacterial live vectors such as *Shigella* encoding ETEC CFs [8], multiple epitope fusion antigens [9], and ST toxoids [10]. Stimulating intestinal secretory IgA antibodies that bind CFs and prevent ETEC from attaching to human small intestine mucosa is generally considered to be fundamental to a successful ETEC vaccine, although some contend that parenteral vaccine-induced serum IgG antibodies that transude onto intestinal mucosa may also prevent diarrhea in humans caused by bacterial enteropathogens [11]. Most current ETEC vaccines contain a mix of antigens directed against CFA/I, CFA/II and CFA/IV antigens.

Minor putative CFs also exist for which data supporting their role in pathogenesis in humans is less compelling or lacking, although they also mediate attachment to human cells in tissue culture. Possible exceptions are CS17 LT-only strains that evoked diarrhea in challenged volunteers [12] and LT-only isolates encoding CS7 that were incriminated in small cohort studies of pediatric diarrhea [13,14]. While minor CF antigens CS7, CS12, CS14, CS17, CS18, CS19, CS20, CS21 and CS30 have received most attention, others have also been described including CS8, CS10, CS11, CS13, CS15 and CS23 [15–19].

DNA-based high-throughput diagnostics have enabled large epidemiologic studies to quantify the ETEC disease burden among young children in developing countries and to assess the prevalence of various CFs. The overall objective of the Global Enteric Multicenter Study (GEMS) was to estimate the population-based burden, microbiologic etiology and adverse clinical consequences of moderate-to-severe diarrhea among children 0–59 months of age in study sites in sub-Saharan Africa and South Asia to guide the development and implementation of vaccines and other interventions [1]. GEMS tested for a large number of diarrheal pathogens, including ETEC, among cases of moderate-to-severe diarrhea (MSD) and matched (by age, gender, neighborhood and time of presentation) control children without diarrhea in three age strata (0–11, 12–23 and 24–59 months) at four sites in sub-Saharan Africa and three in South Asia [1,20], the geographic regions where 80% of global diarrheal deaths occur. The main underlying assumption of GEMS was that a limited number of etiologic agents may be responsible for a disproportionately large fraction of all MSD [21]. ST-producing ETEC, i.e., LT/ST and ST-only strains, were significantly incriminated as pathogens and placed ETEC as one of the top four pathogens associated with MSD across all seven sites and age groups [1].

A secondary aim of GEMS was to elucidate the proportion of ETEC strains, by toxin genotype that encode the main CFs and selected minor CFs. Herein we present the proportion of GEMS ETEC isolates that encode the main CFs found in most ETEC vaccines under development, and the prevalence of ten other putative attachment factors (CS7, CS12, CS13, CS14, CS17, CS18, CS19, CS20, CS21, CS30) that have been proposed as potential antigens to broaden ETEC vaccine immunoprophylaxis. In addition, based on the GEMS case/control design, we utilized conditional logistic regression to assess the strength of association with MSD of ETEC of the different toxin genotypes encoding the major and minor CFs.

## Methods

### Study design and population

The rationale [20], assumptions, clinical, epidemiological and microbiological methods of GEMS [1,22], a three-year case-control study undertaken among children <5 years of age in Gambia (Basse), Mali (Bamako), Mozambique (Manhiça) and Kenya (Siaya County) in sub-Saharan Africa and India (Kolkata), Bangladesh (Mirzapur) and Pakistan (Karachi-Bin Qasim Town) in South Asia, have been described. MSD was defined as an acute episode of diarrhea (≥3 loose stools during a 24-hour period) that started within the previous seven days, was separated from another episode by ≥7 days, and was accompanied by either signs of dehydration (sunken eyes, slow abdominal “skin pinch” recoil or administration of intravenous fluids), dysentery or admission to hospital based on clinical concern over diarrheal disease severity [1,23].

The current GEMS report includes a descriptive summary of the prevalence of CFs among ETEC isolates from cases and controls by toxin profile and country, followed by analyses that utilize the GEMS matched case-control design to test hypotheses that major or minor CFs might be significantly related to the risk of MSD. Collectively, this information can help guide ETEC vaccine developers.

### Ethics

This research involved characterization of isolates of enterotoxigenic *Escherichia coli* obtained from participants in the Global Enteric Multicenter Study (GEMS). The ethical review methods for this study were described in detail [23], as well as summarized in the overall publication of the results of the clinical study "The clinical protocol was approved by ethics committees at the University of Maryland, Baltimore, MD, USA, and at every field site [1]. Written informed consent was obtained from the parent or primary caretaker of each participant before initiation of study activities [1].” The clinical protocol included the collection of stool specimens or rectal swabs that were tested for the presence of colonies of enterotoxigenic *E*. *coli* and for the presence of other enteric pathogens [1,22].

### Procedures

Stools/rectal swabs from cases and controls were cultured onto MacConkey and xylose/lysine/ deoxycholate agar and three *E*. *coli* colonies per subject were identified and pooled for extraction of DNA which was then tested by a multiplex PCR containing primers to amplify *eltB* (LT B subunit) and *est* (ST) [22]. ETEC strains were shipped to the University of Chile and confirmed by PCR to detect LT and ST variants (STh and STp) [24,25]. Confirmed ETEC isolates were further tested by monoplex or multiplex PCRs using primers that detect target genes encoding the major CFs (CFA/I, CFA/II [CS1, CS2, CS3], CFA/IV [CS4, CS5, CS6]) [16,24,26,27] and various minor CFs (CS7, CS12, CS13, CS14, CS17, CS18, CS19, CS20, CS21, CS30) (**Table 1**) [16–19,28].

**Table 1:** Primers used in this study for detection of toxin and colonization factor genes in ETEC strains.

Gene (Toxin/CFs)	Primer sequence (5’– 3’)	Concentration (pmol/uL)	PCR type (Mn/Mt)	Product size (bp)	Reference
*eltB* (LT)	F: GCACACGGAGCTCCTCAGT	0.2	Mn	218	Vidal *et al*.[24]
	R: TCCTTCATCCTTTCAATGGCTTT				
*sta2* (STh)	F: TTCTTTCTGTATTGTCTTTTTCACC	0.2	Mn	193	Vidal *et al*.[24]
	R: TAATAGCACCCGGTACAAGCAG				
*sta1* (STp)	F: CCTCGACATATAACATGATGCAACTC	0.2	Mn	127	This study
	R: AAATTGCCAACATTAGCTTTTTCA				
*sta1* (STp)	F: TCTTTCCCCTCTTTTAGTCAG	0.2	Mn	166	Rodas *et al*.[25]
	R: ACAGGCAGGATTACAACAAAG				
*cfaB* (CFA/I)	F: ACTATTGGTGCAATGGCTCTGAC	0.2	Mt1	497	Vidal *et al*.[24]
	R: CAGGATCCCAAAGTCATTACAAG				
*cooA* (CS1)	F: GAGAAGACCATTAGCGTTACGG	0.16	Mt3	410	Vidal *et al*.[24]
	R: CCCTGATATTGACCAGCTGTTAG				
*cotA* (CS2)	F: ACTGTAACTGCTAGCGTTGATCC	0.2	Mt1	358	Vidal *et al*.[24]
	R: TGCTTCCTGCATTAATAACGAGT				
*cstH* (CS3)	F: CCCACTCTAACCAAAGAACTGG	0.48	Mt3	300	Vidal *et al*.[24]
	R: CGTATTTCCAGCATTTTTATCCA				
*csaB* (CS4)	F: ATTGATATTTTGCAAGCTGATGG	0.32	Mt3	242	Vidal *et al*.[24]
	R: GTCACATCTGCGGTTGATAGAGT				
*csfA* (CS5)	F: TCCGCTCCCGTTACTCAG	0.2	Mt2	226	Sjöling *et al*.[27]
	R: GAAAAGCGTTCACACTGTTTATATT				
*cssA* (CS6)	F: AAATGTATCCCAGGTAACGGTCT	0.2	Mt2	165	Vidal *et al*.[24]
	R: TGTTGATTAGGCGTAACCTCTGT				
*csvA* (CS7)	F: TGCTCCCGTTACTAAAAATAC	0.16	Mt4	203	Del Canto *et al*.[16]
	R: TAGATGTCGTATCACTACGT				
*cswA* (CS12)	F: GCGAATAACAATGATGCAAG	0.16	Mt4	263	Del Canto *et al*.[16]
	R: CCTGACTGGTTTACAAGATA				
*cshE* (CS13)	F: GGGACTGCCACAATGAATTT	0.4	Mn	178	Sjöling *et al*.[27]
	R: CAGCACCACCTGCTGATTTA				
*csuA1* (CS14)	F: TTTGCAACCGACATCTACCA	0.4	Mn	162	Sjöling *et al*.[27]
	R: CCGGATGTAGTTGCTCCAAT				
*csbA-csdA* (CS17-19)	F: TAAACTTGATCTTCTGCAAGC	0.16	Mt4	348	Del Canto *et al*.[16]
	R: GCATGAATCGTAAGCTGTTG				
*csbA* (CS17)	F: TAAACTTGATCTTCTGCAAGC	0.16	Mn	324	Del Canto *et al*.[16]
	R: TCAGGCGCAGTTCCTTGTGTG				
*fotG* (CS18)	F: ATCCGTCAGGTGTTTGTGGT	0.4	Mn	362	Rodas *et al*.[25]
	R: CACCTGAATTCCTCGACAGG				
*csnA* (CS20)	F: AGGTATCCAAATCCGCACTG	0.4	Mn	114	Sjöling *et al*.[27]
	R: CATCAGCCAGCACATAGGAA				
*fotA* (CS18)	F: TGGTGTAGGTGTGTTTGTCC	0.2	Mn	193	This study
	R: AGTACCAGCTTTAACCTGACC				
*csnA* (CS20)	F: CCTGATTAACTGTGACAGCCT	0.2	Mn	189	This study
	R: ACAACGTCAAGTTTTTGATCGC				
*lngA* (CS21)	F: TCATGAGCCTGCTGGAAGTTATCA	0.16	Mn	617	Pichel *et al*.[28]
	R: TCCGGCTACCTAAAGTAATTGAGT				
*csmA* (CS30)	F: AGTCAGCTCTTGCAGCCAGT	0.2	Mn	219	von Mentzer *et al*.[19]
	R: CCTTGGTACCATTGCTGGTT				

CFs: Colonization Factors Mn: Monoplex PCR Mt: Multiplex-PCR. The numbers associated with each Mt correspond to the primers associated with each multiplex reaction.

The rationale for selecting some of the minor CFs for testing was because epidemiologic data incriminate them as being associated with pediatric diarrhea (e.g., LT-only strains expressing CS7) [13,14]. We tested for other minor CFs because volunteer challenges with well characterized strains encoding them showed that they can elicit diarrhea (e.g., CS17 and CS19) [29]. CS14 was studied because it has been common among ST-only and LT/ST ETEC in various reports [30,31]. CS18 and CS20 were studied because they share high homology. CS12 and CS21 (“longus”) were studied because of long-term interest of some GEMS investigators [32–34], and their global prevalence [30], and advocates contending that they are virulence attributes [35]. CS30 was studied because it is found in LT/STp isolates and has homology to CS18 and CS20 [19].

We also selected the cited minor CFs to be studied based on their genetic relationships within the usher genomic typing system [36–38]. The majority of ETEC CFs are synthesized and transported utilizing a chaperone-usher system that typically contains four genes encoding a periplasmic chaperone, a major fimbrial subunit, an outer membrane usher and a minor subunit tip adhesin. Since there is only a single usher gene among these ETEC CFs, they can be readily classified by their sequences [36–38]. All the major CFs except CS3 and CS6 are found within the α usher sequence group, including CFA/I, CS1, CS2, CS4 and CS5. Minor CFs in this α group include CS7, CS14, CS17 and CS19; these homologies were another reason we tested for these CFs among the GEMS ETEC isolates. The γ_2_ usher family includes four minor CFs of interest, CS12, CS18, CS20 and CS30, which is partly why we tested for them. CS13 belongs to the κ group [37]. CS3 and CS6 major CFs reside within the γ_3_ usher group. CS8 (previously called CFA/III), which was not studied, and CS21 are not classifiable within the chaperone-usher system, since they are synthesized as type IVb pili.

**Fig 1:**
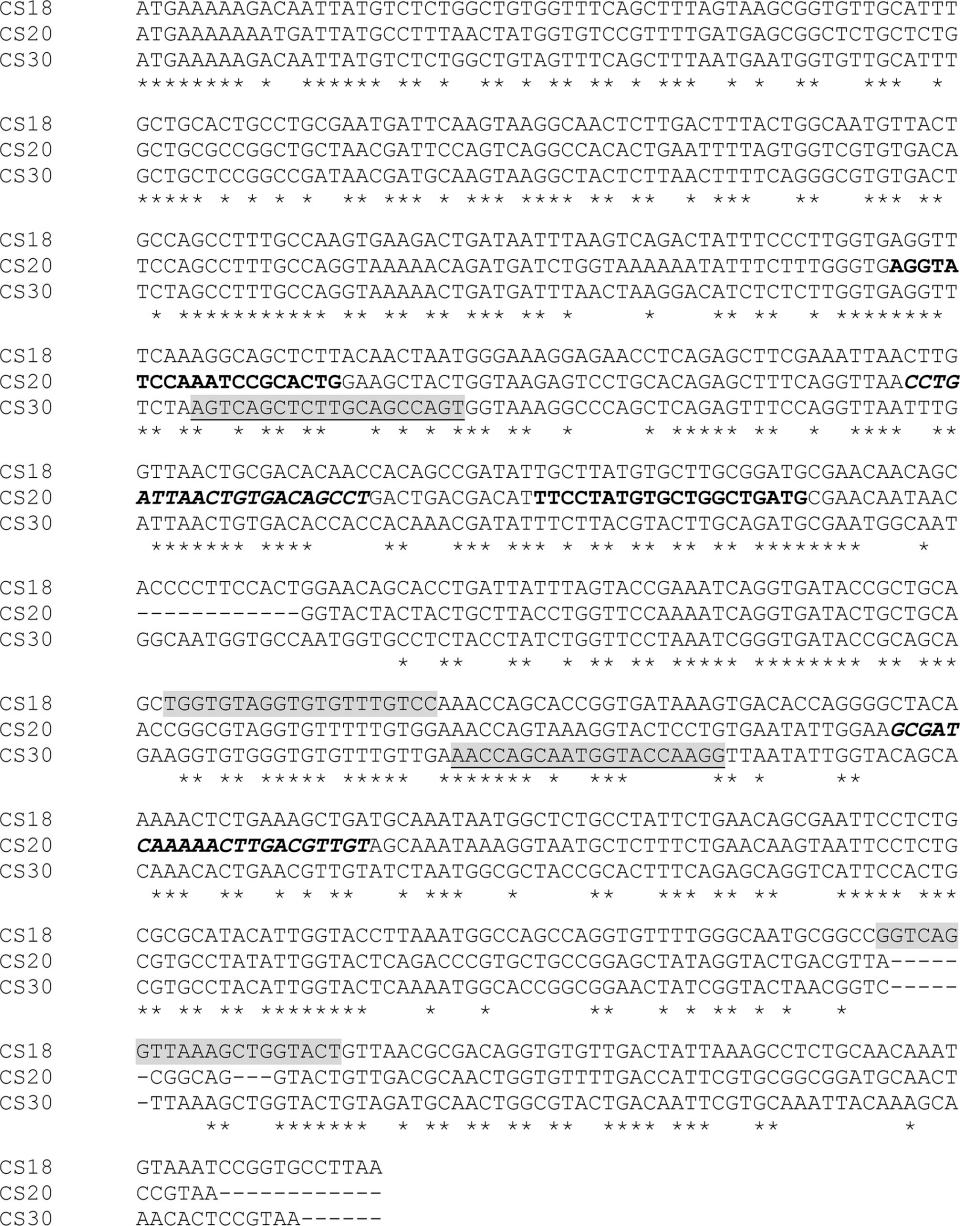
Alignments of the major structural subunit genes *fotA* (CS18), *csnA* (CS20) and *csmA* (CS30). Primer target sequences CS18-F2 and CS18-R2 are highlighted in light grey. Primer target sequences CS20-F and CS20-R are shown in bold. Primer target sequences CS20-F2 and CS20-R2 are shown in bold italics. Primer target sequences for-*csmA* and rev-*csmA* are highlighted in light grey and underlined.

**Fig 2:**
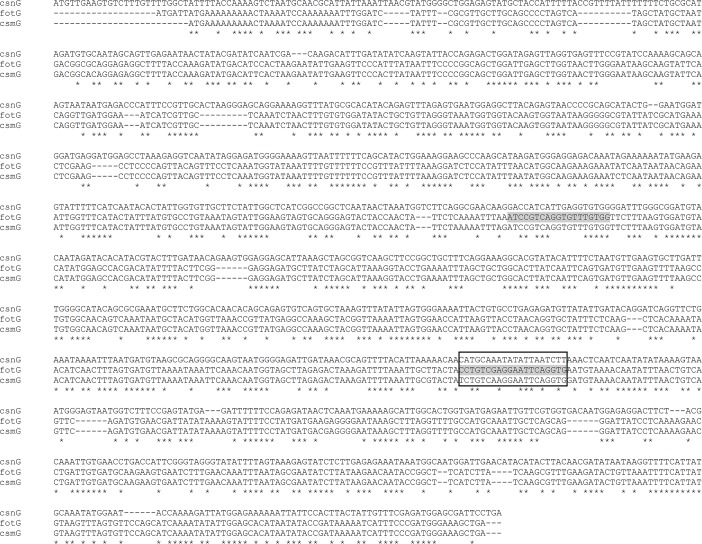
Minor structural subunit genes *fotG* (CS18), *csnG* (CS20) and *csmG* (CS30). Primers CS18-F and CS18-R that allow detection of *fotG* are highlighted in light grey.

CS18 and CS20 were initially tested using previously described primers that amplify sequences within *fotG* (which encodes the tip adhesin of CS18) [25], and *csnA* (which encodes the major subunit of CS20) [27]. With the recent report of CS30, a new minor colonization factor (CF) [19], and revelation of its similarity to CS18 and CS20, new primers were designed to enhance specificity. The new primers to detect CS18 amplify a sequence within *fotA* (that encodes the major fimbrial subunit) rather than *fotG*. Alignments of major and minor structural subunit genes of CS18, CS20 and CS30 are shown in **Figs 1 & 2.** Reference strains served as positive controls [16].

All isolates from the 806 cases and 711 control participants whose cultures yielded ETEC isolates were also tested by polymerase chain reaction (PCR) for genes encoding CFA/I and CS1-CS6, the major colonization factors (CFs). In addition, these isolates were all tested by PCR for several minor CFs including CS7, CS12, CS14, CS17 and CS21, all of which had been proposed to be potential virulence attributes and potential antigens to be included in an ETEC vaccine intending to elicit anti-colonization immunity.

ETEC isolates from cases (N = 203) and controls (N = 295) that were negative for the major CFs and for minor CFs CS7, CS12, CS14, CS17 and CS21 were tested for genes encoding several additional minor CFs including CS13, CS18, CS19 and CS20; isolates from nine cases and eight controls could not be tested because they were not recoverable. After completion of testing for CS13, CS18, CS19 and CS20, a new minor CF, CS30, was reported as being found among a proportion of LT/ST isolates [19]. We thereupon re-tested for CS30 the 113 LT/ST isolates that were among the above-mentioned 203 case isolates; the 65 LT/ST isolates among the above-mentioned 295 control isolates were also tested. However, because of sequence homologies among CS30, CS18 and CS20, we also re-tested the 65 case and 113 control isolates for CS18 and CS20 using new primers that were designed to increase specificity (*vide supra*) (**Figs 1 & 2**).

Crude bacterial lysate was obtained by boiling five pooled colonies of each ETEC isolate in 0·1% Triton X-100 for 10 min, followed by centrifugation at 8000×g for five minutes to separate template DNA in the supernatant from cellular debris. PCR was performed with total bacterial DNA in a 25-μL reaction, containing 10 mmol/L deoxyribonucleotide triphosphate mix, 30 mmol/L MgCl_2_, 1× reaction buffer (10 mmol/L Tris–HCl, 50 mmol/L KCl), one Unit of Taq polymerase (GoTaq; Promega, Madison, WI), and 1 μL of template DNA. Primers were used at concentrations shown in **Table 1**. To prevent nonspecific amplification, we used the “hot start” technique, which includes preheating reaction mixtures to 94°C for five minutes before adding Taq DNA polymerase. Samples were amplified for 35 cycles, with each cycle comprising 90 seconds at 94°C for denaturation, 30 seconds at specific primers annealing temperatures, 60 seconds at 68°C for strand elongation, and a final extension at 72°C for five minutes. PCR products were electrophoresed in 2.0% agarose, stained with ethidium bromide, and amplicons identified based on expected size of the amplified product compared with amplicons of reference strains.

A subset of ETEC isolates were sent to the WHO Enterotoxigenic *Escherichia coli* Reference Laboratory, University of Gothenburg, Sweden, where they were tested for STp, STh, major CFs and phenotypic expression of CFs using monoclonal antibodies [27]. Gothenburg primers for STp and CS5 were used in Chile in addition to local primers [16,24,25,27].

### Data analysis

Presentation of the descriptive observational data and analyses were restricted to ETEC cases that had a single ETEC toxin/CF genotype pattern.

#### Descriptive data

Prevalences of ETEC CFs were expressed as percentages in a stratified manner by ETEC toxin profile, site and region.

#### Matched case-control studies of the associations between ETEC CFs and MSD

Analyses of the strength of association between ETEC toxin and CF genotypes and MSD were performed using conditional logistic regression models in which the outcome was case-control status (MSD) and the independent variable (covariate) was whether the child’s ETEC had the specific CFA (no/yes) [39], while applying Firth’s penalized likelihood approach [40]. Use of conditional logistic regression was dictated by the matched case/control design, while the Firth approach was indicated because the subset of ETEC cases and ETEC controls that encode CFs is relatively small compared to the total number of children with ETEC infection. Matched odds ratios (ORs) and corresponding 95% confidence intervals (CIs) were obtained from these models. Because pooled as well as site-specific analyses were conducted, we examined for heterogeneity in ORs across sites using Chi square test for heterogeneity. A p ≤0.05 was considered significant. We did not use a Bonferroni adjustment for these 19 individual conditional logistic regression analyses of the association of individual minor CFs with MSD, as in each instance an individual hypothesis was tested [41–44]. Data were analyzed using SPSS version 23 (IBM, Inc., Armonk, NY) and SAS statistical software version 9.4 (SAS Institute Inc. Cay, NC, USA).

## Results

When tested in a standardized manner in GEMS field-site laboratories using a multiplex PCR that included primers to detect genes encoding STh and LT [22], colonies from 1067 of 9439 MSD cases (11.3%) and from 975 of 13,129 matched control subjects (7.4%) tested positive. ETEC isolates were sent to the GEMS Reference Laboratory at the University of Chile to detect CFs [26]. Upon arrival, all isolates were re-tested to detect LT, STh and STp genes, since upon storage, sub-culture or transport, ETEC isolates may lose toxin or CF genes [45–48]. *E*. *coli* isolates from 894 of the 1067 cases were confirmed as ETEC and among the triplets of isolates tested from each case, 806 cases (90.2%) had a single toxin/CF profile observed; 83 others (9.6%) had two profiles and five cases (0.6%) had three different profiles recorded. A single toxin/CF profile was found among triplets of 711 controls.

### Toxin genotypes among ETEC isolates from MSD cases and controls

Among the 806 single toxin/CF profile cases and 711 controls, the percentages of children at each site who harbored ETEC isolates of the different enterotoxin genotypes are shown in **Table 2**, revealing the relative frequency of LT-only, STh-only, STp-only, LT/STh, and LT/STp infections.

**Table 2:** Toxin profile of ETEC isolates from cases and from controls by continent and across all seven GEMS sites combined.

	Africa		Asia		Asia & Africa	
Toxin Profile	Cases (N = 510)	Controls (N = 480)	Cases (N = 296)	Controls (N = 231)	Cases (*N = 806*)	Controls (*N = 711*)
LT-only	171 (33.5%)*	249 (51.9%)^+^	85 (28.7%)^#^	84 (36.4%)^$^	*256 (31*.*8%)*	*333 (46*.*8%)*
Any ST-only	182 (35.7%)*	98 (20.4%)^+^	109 (36.8%)^#^	48 (20.8%)^$^	*291 (36*.*1%)*	*146 (20*.*5%)*
STh-only	177 (34.7%)*	94 (19.6%)^+^	107 (36.1%)^#^	40 (17.3)^$^	*284 (35*.*2%)*	*134 (18*.*8%)*
STp-only	5 (1.0%)*	4 (0.8%)^+^	2 (0.7%)^#^	7 (3.0%)^$^	*7 (0*.*9%)*	*11 (1*.*5%)*
STh/STp	0 (0%)*	0 (0%)^+^	0 (0%)^#^	1 (0.4%)^$^	*0 (0%)*	*1 (0*.*1%)*
All LT/ST	157 (30.8%)*	133 (27.7%)^+^	102 (34.5%)^#^	99 (42.9%)^$^	*259 (32*.*1%)*	*232 (32*.*6%)*
LT/STh	104 (20.4%)*	65 (13.5%)^+^	74 (25.0%)^#^	30 (13.0%)^$^	*178 (22*.*1%)*	*95 (13*.*4%)*
LT/STp	53 (10.4%)*	67 (14.0%)^+^	27 (9.1%)^#^	69 (29.9%)^$^	*80 (9*.*9%)*	*136 (19*.*1%)*
LT/STh/STp	0 (0%)*	1 (0.2%)^+^	1 (0.3%)^#^	0 (0%)^$^	*1 (0*.*1%)*	*1 (0*.*1%)*
All ST-only ^+^ all LT/ST	339 (66.5%)*	231 (48.1%)^+^	211 (71.3%)^#^	147 (63.6%)^$^	*550 (68*.*2%)*	*378 (53*.*2%)*

Data presented are the number of ETEC strains (and percentages) with the indicated toxin profile

* Percent of 510

^+^ Percent of 480

^#^ percent of 296

^$^ Percent of 231

Overall, 68.2% of isolates from cases (N = 550) were either ST-only (N = 291, 36.1%) or LT/ST (N = 259, 32.1%), the genotypes strongly associated with MSD in GEMS [1]. STh-only strains were isolated from 284 (35.2%) of 806 cases. The remaining case isolates (N = 256, 31.8%) were LT-only.

### Major CFs among ETEC from MSD cases and controls

**Fig 3:**
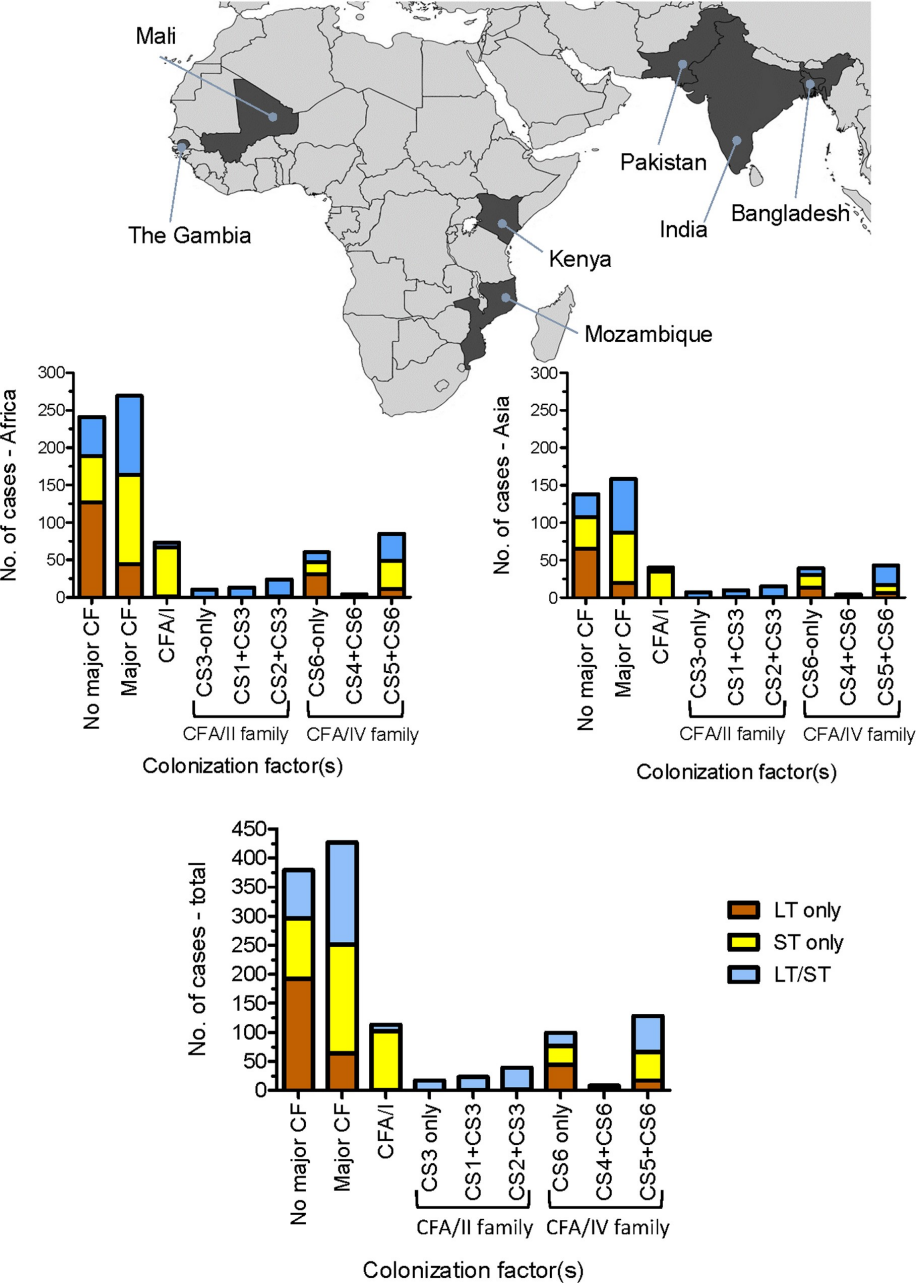
The prevalence of ETEC isolates from cases of moderate-to-severe diarrhea encoding both LT and ST enterotoxins (blue), ST-only (yellow) or LT-only (blue) and also encoding the major colonization factors CFA/I, CFA/II or CFA/IV. Data from the four African, three Asian and all sites are shown.

The proportion of ETEC strains from MSD cases that carry major CF antigens including CFA/I and CS1-CS6, by toxin genotype, are shown by country (**Table 3**) and summarized by continent (**Fig 3**).

**Table 3:** The prevalence of major colonization factors that are contained in leading vaccine candidates, by toxin profiles, among ETEC strains from 806 MSD cases from individual GEMS sites in Africa and Asia.

Site	Toxin profile	Total cases	CFA/I	Any CFA/II^a^	CS3-only	CS1+CS3	CS2+CS3	Any CFA/IV^b^	CS6-only	CS4+CS6	CS5+CS6
Gambia	LT-only	35	0/35 (0%)	1/35 (2.9%)	0/35 (0%)	0/35 (0%)	1/35 (2.9%)	7/35 (20.0%)	5/35 (14.3%)	0/35 (0%)	2/35 (5.7%)
	ST-only	46	16/46 (34.8%)	0/46 (0%)	0/46 (0%)	0/46 (0%)	0/46 (0%)	17/46 (37.0%)	2/46 (4.3%)	0/46 (0%)	15/46 (32.6%)
	LT/ST	39	0/39 (0%)	14/39 (35.9%)	6/39 (15.4%)	1/39 (2.6%)	7/39 (17.9%)	14/39 (35.9%)	4/39 (10.3%)	0/39 (0%)	10/39 (25.6%)
	ST+LT/ST	85	16/85 (18.8%)	14/85 (16.5%)	6/85 (7.1%)	1/85 (1.2%)	7/85 (8.2%)	31/85 (36.5%)	6/85 (7.1%)	0/85 (0%)	25/85 (29.4%)
Mali	LT-only	51	1/51 (2.0%)	0/51 (0%)	0/51 (0%)	0/51 (0%)	0/51 (0%)	15/51 (29.4%)	14/51 (27.5%)	0/51 (0%)	1/51 (2.0%)
	ST-only	46	17/46 (37.0%)	0/46 (0%)	0/46 (0%)	0/46 (0%)	0/46 (0%)	20/46 (43.5%)	4/46 (8.7%)	0/46 (0%)	16/46 (34.8%)
	LT/ST	41	4/41 (9.8%)	6/41 (14.6%)	1/41 (2.4%)	1/41 (2.4%)	4/41 (9.8%)	12/41 (29.3%)	1/41 (2.4%)	3/41 (7.3%)	8/41 (19.5%)
	ST+LT/ST	87	21/87 (24.1%)	6/87 (6.9%)	1/87 (1.1%)	1/87 (1.1%)	4/87 (4.6%)	32/87 (36.8%)	5/87 (5.7%)	3/87 (3.4%)	24/87 (27.6%)
Mozambique	LT-only	16	0/16 (0%)	0/16 (0%)	0/16 (0%)	0/16 (0%)	0/16 (0%)	4/16 (25.0%)	1/16 (6.3%)	0/16 (0%)	3/16 (18.8%)
	ST-only	22	9/22 (40.9%)	0/22 (0%)	0/22 (0%)	0/22 (0%)	0/22 (0%)	4/22 (18.2%)	2/22 (9.1%)	0/22 (0%)	2/22 (9.1%)
	LT/ST	25	0/25 (0%)	8/25 (32.0%)	1/25 (4.0%)	2/25 (8.0%)	5/25 (20.0%)	13/25 (52.0%)	1/25 (4.0%)	0/25 (0%)	12/25 (48.0%)
	ST+LT/ST	47	9/47 (19.1%)	8/47 (17.0%)	1/47 (2.1%)	2/47 (4.3%)	5/47 (10.6%)	17/47 (36.2%)	3/47 (6.4%)	0/47 (0%)	14/47 (29.8%)
Kenya	LT-only	69	0/69 (0%)	0/69 (0%)	0/69 (0%)	0/69 (0%)	0/69 (0%)	16/69 (23.2%)	11/69 (15.9%)	0/69 (0%)	5/69 (7.2%)
	ST-only	68	24/68 (35.3%)	0/68 (0%)	0/68 (0%)	0/68 (0%)	0/68 (0%)	13/68 (19.1%)	3/68 (4.4%)	0/68 (0%)	10/68 (14.7%)
	LT/ST	52	2/52 (3.8%)	18/52 (34.6%)	2/52 (3.8%)	9/52 (17.3%)	7/52 (13.5%)	14/52 (26.9%)	2/52 (3.8%)	1/52 (1.9%)	11/52 (21.2%)
	ST+LT/ST	120	26/120 (21.7%)	18/120 (15.0%)	2/120 (1.7%)	9/120 (7.5%)	7/120 (5.8%)	27/120 (22.5%)	5/120 (4.2%)	1/120 (0.8%)	21/120 (17.5%)
***Africa***	***LT-only***	***171***	***1/171 (0*.*6%)***	***1/171 (0*.*6%)***	***0/171 (0%)***	***0/171 (0%)***	***1/171 (0*.*6%)***	***42/171 (24*.*6%)***	***31/171 (18*.*1%)***	***0/171 (0%)***	***11/171 (6*.*4%)***
	***ST-only***	***182***	***66/182 (36*.*3%)***	***0/182 (0%)***	***0/182 (0%)***	***0/182 (0%)***	***0/182 (0%)***	***54/182 (29*.*7%)***	***11/182 (6*.*0%)***	***0/182 (0%)***	***43/182 (23*.*6%)***
	***LT/ST***	***157***	***6/157 (3*.*8%)***	***46/157 (29*.*3%)***	***10/157 (6*.*4%)***	***13/157 (8*.*3%)***	***23/157 (14*.*6%)***	***53/157 (33*.*8%)***	***8/157 (5*.*1%)***	***4/157 (2*.*5%)***	***41/157 (26*.*1%)***
	***ST+LT/ST***	***339***	***72/339 (21*.*2%)***	***46/339 (13*.*6%)***	***10/339 (2*.*9%)***	***13/339 (3*.*8%)***	***23/339 (6*.*8%)***	***107/339 (31*.*6%)***	***19/339 (5*.*6%)***	***4/339 (1*.*2%)***	***84/339 (24*.*8%)***
India	LT-only	26	0/26 (0%)	0/26 (0%)	0/26 (0%)	0/26 (0%)	0/26 (0%)	8/26 (30.8%)	5/26 (19.2%)	0/26 (0%)	3/26 (11.5%)
	ST-only	36	13/36 (36.1%)	0/36 (0%)	0/36 (0%)	0/36 (0%)	0/36 (0%)	8/36 (22.2%)	4/36 (11.1%)	1/36 (2.8%)	3/36 (8.3%)
	LT/ST	44	3/44 (6.8%)	19/44 (43.2%)	6/44 (13.6%)	6/44 (13.6%)	7/44 (15.9%)	13/44 (29.5%)	1/44 (2.3%)	0/44 (0%)	12/44 (27.3%)
	ST+LT/ST	80	16/80 (20.0%)	19/80 (23.8%)	6/80 (7.5%)	6/80 (7.5%)	7/80 (8.8%)	21/80 (26.3%)	5/80 (6.3%)	1/80 (1.3%)	15/80 (18.8%)
Bangladesh	LT-only	17	0/17 (0%)	0/17 (0%)	0/17 (0%)	0/17 (0%)	0/17 (0%)	2/17 (11.8%)	1/17 (5.9%)	0/17 (0%)	1/17 (5.9%)
	ST-only	15	5/15 (33.3%)	1/15 (6.7%)	0/15 (0%)	0/15 (0%)	1/15 (6.7%)	5/15 (33.3%)	4/15 (26.7%)	0/15 (0%)	1/15 (6.7%)
	LT/ST	24	1/24 (4.2%)	5/24 (20.8%)	0/24 (0%)	3/24 (12.5%)	2/24 (8.3%)	7/24 (29.2%)	1/24 (4.2%)	0/24 (0%)	6/24 (25.0%)
	ST+LT/ST	39	6/39 (15.4%)	6/39 (15.4%)	0/39 (0%)	3/39 (7.7%)	3/39 (7.7%)	12/39 (30.8%)	5/39 (12.8%)	0/39 (0%)	7/39 (17.9%)
Pakistan	LT-only	42	0/42 (0%)	1/42 (2·4%)	0/42 (0%)	1/42 (2.4%)	0/42 (0%)	9/42 (21.4%)	6/42 (14.3%)	0/42 (0%)	3/42 (7.1%)
	ST-only	58	17/58 (29.3%)	0/58 (0%)	0/58 (0%)	0/58 (0%)	0/58 (0%)	18/58 (31.0%)	8/58 (13.8%)	2/58 (3.4%)	8/58 (13.8%)
	LT/ST	34	1/34 (2.9%)	6/34 (17.6%)	1/34 (2.9%)	0/34 (0%)	5/34 (14.7%)	16/34 (47.1%)	1/34 (2.9%)	1/34 (2.9%)	14/34 (41.2%)
	ST+LT/ST	92	18/92 (19.6%)	6/92 (6.5%)	1/92 (1.1%)	0/92 (0%)	5/92 (5.4%)	34/92 (37.0%)	9/92 (9.8%)	3/92 (3.3%)	22/92 (23.9%)
***Asia***	***LT-only***	***85***	***0/85 (0%)***	***1/85 (1*.*2%)***	***0/85 (0%)***	***1/85 (1*.*2%)***	***0/85 (0%)***	***19/85 (22*.*4%)***	***12/85 (14*.*1%)***	***0/85 (0%)***	***7/85 (8*.*2%)***
	***ST-only***	***109***	***35/109 (32*.*1%)***	***1/109 (0*.*9%)***	***0/109 (0%)***	***0/109 (0%)***	***1/109 (0*.*9%)***	***31/109 (28*.*4%)***	***16/109 (14*.*7%)***	***3/109 (2*.*8%)***	***12/109 (11*.*0%)***
	***LT/ST***	***102***	***5/102 (4*.*9%)***	***30/102 (29*.*4%)***	***7/102 (6*.*9%)***	***9/102 (8*.*8%)***	***14/102 (13*.*7%)***	***36/102 (35*.*3%)***	***3/102 (2*.*9%)***	***1/102 (1*.*0%)***	***32/102 (31*.*4%)***
	***ST+LT/ST***	***211***	***40/211 (19*.*0%)***	***31/211 (14*.*8%)***	***7/211 (3*.*3%)***	***9/211 (4*.*3%)***	***15/211 (7*.*1%)***	***67/211 (31***.***8%)***	***19/211 (9*.*0%)***	***4/211 (1*.*9%)***	***44/211 (20*.*9%)***
***Asia & Africa***	***LT-only***	***256***	***1/256 (0*.*4%)***	***2/256 (0*·*8%)***	***0/256 (0%)***	***1/256 (0*.*4%)***	***1/256 (0*.*4%)***	***61/256 (23*.*8%)***	***43/256 (16*.*8%)***	***0/256 (0%)***	***18/256 (7*.*0%)***
	***ST-only***	***291***	***101/291 (34*.*7%)***	***1/291 (0*·*3%)***	***0/291 (0%)***	***0/291 (0%)***	***1/291 (0*.*3%)***	***85/291 (29*.*2%)***	***27/291 (9*.*3%)***	***3/291 (1*.*0%)***	***55/291 (18*.*9%)***
	***LT/ST***	***259***	***11/259 (4*.*2%)***	***76/259 (29*.*3%)***	***17/259 (6*.*6%)***	***22/259 (8*.*5%)***	***37/259 (14*.*3%)***	***89/259 (34*.*4%)***	***11/259 (4*.*2%)***	***5/259 (1*.*9%)***	***73/259 (28*.*2%)***
	***ST+LT/ST***	***550***	***112/550 (20*.*4%)***	***77 /550 (14*.*0%)***	***17/550 (3*.*1%)***	***22/550 (4*.*0%)***	***38/550 (6*.*9%)***	***174/550 (31*.*6%)***	***38/550 (6*.*9%)***	***8/550 (1*.*5%)***	***128/550 (23*.*3%)***

^**a**^ CFA/II strains are defined as encoding CS3 either alone or in combination with either CS1 or CS2 but never both CS1 and CS2. Very rarely isolates that encode CS1 without CS3 have been reported.[26] The rare CFs of this nature recovered in GEMS are not included in this table.

^**b**^ CFA/IV strains are defined as encoding CS6 either alone or in combination with either CS4 or CS5, but never both CS4 and CS5. Very rarely isolates that encode CS5 without CS6 have been reported. The few such isolates recovered in GEMS are not included in this table.

Overall, 363 (66.0%) of 550 ST-only and LT/ST strains encoded a major CF including 20.4% encoding CFA/I, 14.0% encoding CFA/II (i.e., CS3 alone or with CS1 or CS2) and 31.6% encoding CFA/IV (i.e., CS6 alone or with CS4 or CS5). The only major CF commonly observed among LT-only isolates was CS6-only, recorded in 43 of 256 LT-only strains (16.8%). Only three of 256 LT-only strains (1.2%) encoded CFA/I or CFA/II.

The 975 putative ETEC strains from control subjects that arrived at the GEMS ETEC Reference Laboratory at the University of Chile were re-tested to detect LT, STh and STp genes, of which 748 were confirmed as positive. **Table 4** summarizes the proportion of ETEC strains from controls that encoded the major CF antigens including CFA/I and CS1-CS6, with data presented by country, continent and toxin genotype. Overall, 170 of 378 ST-only and LT/ST strains (45.0%) encoded a major CF antigen including 6.9% encoding CFA/I, 15.1% encoding CFA/II and 23.0% encoding CFA/IV. Among 333 LT-only isolates, one (0.6%) encoded CS3-only, 63 encoded CS6-only (18.9%), one encoded CS4+CS6 (0.3%) and 29 had CS5+CS6 (8.7%).

**Table 4:** The prevalence of major colonization factors that are contained in leading vaccine candidates, by toxin profiles, among ETEC strains from 711 control subjects from GEMS sites in Africa and Asia.

Site	Toxin profile	Total controls	CFA/I	Any CFA/II^a^	CS3-only	CS1+CS3	CS2+CS3	Any CFA/IV^b^	CS6-only	CS4+CS6	CS5+CS6
Gambia	LT-only	58	0/58 (0%)	0/58 (0%)	0/58 (0%)	0/58 (0%)	0/58 (0%)	16/58 (27.6%)	12/58 (20.7%)	0/58 (0%)	4/58 (6.9%)
	ST-only	33	8/33 (24.2%)	0/33 (0%)	0/33 (0%)	0/33 (0%)	0/33 (0%)	17/33 (51.5%)	12/33 (36.4%)	0/33 (0%)	5/33 (15.2%)
	LT/ST	39	0/39 (0%)	10/39 (25.6%)	2/39 (5.1%)	0/39 (0%)	8/39 (20.5%)	8/39 (20.5%)	2/39 (5.1%)	0/39 (0%)	6/39 (15.4%)
	ST+LT/ST	72	8/72 (11.1%)	10/72 (13.9%)	2/72 (2.8%)	0/72 (0%)	8/72 (11.1%)	25/72 (34.7%)	14/72 (19.4%)	0/72 (0%)	11/72 (15.3%)
Mali	LT-only	49	0/49 (0%)	0/49 (0%)	0/49 (0%)	0/49 (0%)	0/49 (0%)	19/49 (38.8%)	14/49 (28.6%)	1/49 (2.0%)	4/49 (8.2%)
	ST-only	22	3/22 (13.6%)	0/22 (0%)	0/22 (0%)	0/22 (0%)	0/22 (0%)	6/22 (27.3%)	1/22 (4.5%)	1/22 (4.5%)	4/22 (18.2%)
	LT/ST	26	0/26 (0%)	6/26 (23.1%)	0/26 (0%)	1/26 (3.8%)	5/26 (19.2%)	6/26 (23.1%)	2/26 (7.7%)	0/26 (0%)	4/26 (15.4%)
	ST+LT/ST	48	3/48 (6.3%)	6/48 (12.5%)	0/48 (0%)	1/48 (2.1%)	5/48 (10.4%)	12/48 (25.0%)	3/48 (6.3%)	1/48 (2.1%)	8/48 (16.7%)
Mozambique	LT-only	65	0/65 (0%)	0/65 (0%)	0/65 (0%)	0/65 (0%)	0/65 (0%)	12/65 (18.5%)	6/65 (9.2%)	0/65 (0%)	6/65 (9.2%)
	ST-only	12	0/12 (0%)	0/12 (0%)	0/12 (0%)	0/12 (0%)	0/12 (0%)	2/12 (16.7%)	1/12 (8.3%)	0/12 (0%)	1/12 (8.3%)
	LT/ST	25	0/25 (0%)	8/25 (32.0%)	4/25 (16.0%)	0/25 (0%)	4/25 (16.0%)	7/25 (28.0%)	4/25 (16.0%)	0/25 (0%)	3/25 (12.0%)
	ST+LT/ST	37	0/37 (0%)	8/37 (21.6%)	4/37 (10.8%)	0/37 (0%)	4/37 (10.8%)	9/37 (24.3%)	5/37 (13.5%)	0/37 (0%)	4/37 (10.8%)
Kenya	LT-only	77	0/77 (0%)	0/77 (0%)	0/77 (0%)	0/77 (0%)	0/77 (0%)	20/77 (26.0%)	15/77 (19.5%)	0/77 (0%)	5/77 (6.5%)
	ST-only	31	5/31 (16.1%)	0/31 (0%)	0/31 (0%)	0/31 (0%)	0/31 (0%)	8/31 (25.8%)	5/31 (16.1%)	0/31 (0%)	3/31 (9.7%)
	LT/ST	43	0/43 (0%)	12/43 (27.9%)	3/43 (7.0%)	1/43 (2.3%)	8/43 (18.6%)	8/43 (18.6%)	3/43 (7.0%)	1/43 (2.3%)	4/43 (9.3%)
	ST+LT/ST	74	5/74 (6.8%)	12/74 (16.2%)	3/74 (4.1%)	1/74 (1.4%)	8/74 (10.8%)	16/74 (21.6%)	8/74 (10.8%)	1/74 (1.4%)	7/74 (9.5%)
***Africa***	***LT-only***	***249***	***0/249 (0%)***	***0/249 (0%)***	***0/249 (0%)***	***0/249 (0%)***	***0/249 (0%)***	***67/249 (26*.*9%)***	***47/249 (18*.*9%)***	***1/249 (0*.*4%)***	***19/249 (7*.*6%)***
	***ST-only***	***98***	***16/98 (16*.*3%)***	***0/98 (0%)***	***0/98 (0%)***	***0/98 (0%)***	***0/98 (0%)***	***33/98 (33*.*7%)***	***19/98 (19*.*4%)***	***1/98 (1*.*0%)***	***13/98 (13*.*3%)***
	***LT/ST***	***133***	***0/133 (0%)***	***36/133 (27*.*1%)***	***9/133 (6*.*8%)***	***2/133 (1*.*5%)***	***25/133 (18*.*8%)***	***29/133 (21*.*8%)***	***11/133 (8*.*3%)***	***1/133 (0*.*8%)***	***17/133 (12*.*8%)***
	***ST+LT/ST***	***231***	***16/231 (6*.*9%)***	***36/231 (15*.*6%)***	***9/231 (6*.*8%)***	***2/231 (0*.*9%)***	***25/231 (10*.*8%)***	***62/231 (26*.*8%)***	***30/231 (13*.*0%)***	***2/231 (0*.*9%)***	***30/231 (13*.*0%)***
India	LT-only	35	0/35 (0%)	1/35 (2.9%)	1/35 (2.9%)	0/35 (0%)	0/35 (0%)	9/35 (25.7%)	7/35 (20.0%)	0/35 (0%)	2/35 (5.7%)
	ST-only	15	4/15 (26.7%)	1/15 (6.7%)	0/15 (0%)	0/15 (0%)	1/15 (6.7%)	6/15 (40.0%)	3/15 (20.0%)	0/15 (0%)	3/15 (20.0%)
	LT/ST	22	0/22 (0%)	7/22 (31.8%)	2/22 (9.1%)	3/22 (13.6%)	2/22 (9.1%)	2/22 (9.1%)	0/22 (0%)	0/22 (0%)	2/22 (9.1%)
	ST+LT/ST	37	4/37 (10.8%)	9/37 (21.6%)	2/37 (5.4%)	3/37 (8.1%)	3/37 (8.1%)	8/37 (21.6%)	3/37 (8.1%)	0/37 (0%)	5/37 (13.5%)
Bangladesh	LT-only	25	0/25 (0%)	0/25 (0%)	0/25 (0%)	0/25 (0%)	0/25 (0%)	11/25 (44.0%)	6/25 (24.0%)	0/25 (0%)	5/25 (20.0%)
	ST-only	11	2/11 (18.2%)	0/11 (0%)	0/11 (0%)	0/11 (0%)	0/11 (0%)	3/11 (27.3%)	1/11 (9.1%)	0/11 (0%)	2/11 (18.2%)
	LT/ST	43	0/43 (0%)	8/43 (18.6%)	0/43 (0%)	4/43 (9.3%)	4/43 (9.3%)	3/43 (7.0%)	2/43 (4.7%)	0/43 (0%)	1/43 (2.3%)
	ST+LT/ST	54	2/54 (3.7%)	8/54 (14.8%)	0/54 (0%)	4/54 (7.4%)	4/54 (7.4%)	6/54 (11.1%)	3/54 (5.6%)	0/54 (0%)	3/54 (5.6%)
Pakistan	LT-only	24	0/24 (0%)	0/24 (0%)	0/24 (0%)	0/24 (0%)	0/24 (0%)	6/24 (25.0%)	3/24 (12.5%)	0/24 (0%)	3/24 (12.5%)
	ST-only	22	4/22 (18.2%)	0/22 (0%)	0/22 (0%)	0/22 (0%)	0/22 (0%)	6/22 (27.3%)	3/22 (13.6%)	1/22 (4.5%)	2/22 (9.1%)
	LT/ST	34	0/34 (0%)	5/34 (14.7%)	2/34 (5.9%)	3/34 (8.8%)	0/34 (0%)	5/34 (14.7%)	1/34 (2.9%)	0/34 (0%)	4/34 (11.8%)
	ST+LT/ST	56	4/56 (7.1%)	5/56 (8.9%)	2/56 (3.6%)	3/56 (5.4%)	0/56 (0%)	11/56 (19.6%)	4/56 (7.1%)	1/56 (1.8%)	6/56 (10.7%)
***Asia***	***LT-only***	***84***	***0/84 (0%)***	***1/84 (1*.*2%)***	***1/84 (1*.*2%)***	***0/84 (0%)***	***0/84 (0%)***	***26/84 (31*.*0%)***	***16/84 (19*.*0%)***	***0/84 (0%)***	***10/84 (11*.*9%)***
	***ST-only***	***48***	***10/48 (20*.*8%)***	***1/48 (2*.*1%)***	***0/48 (0*.*9%)***	***0/48 (0%)***	***1/48 (2*.*1%)***	***15/48 (31*.*3%)***	***7/48 (14*.*6%)***	***1/48 (2*.*1%)***	***7/48 (14*.*6%)***
	***LT/ST***	***99***	***0/99 (0%)***	***20/99 (20*.*2%)***	***4/99 (4*.*0%)***	***10/99 (10*.*1%)***	***6/99 (6*.*1%)***	***10/99 (10*.*1%)***	***3/99 (3*.*0%)***	***0/99 (0%)***	***7/99 (7*.*1%)***
	***ST+LT/ST***	***147***	***10/147 (6*.*8%)***	***21/147 (14*.*3%)***	***4/147 (2*.7*%)***	***10/147 (6*.*8%)***	***7/147 (4*.*8%)***	***25/147 (17*.*0%)***	***10/147 (6*.*8%)***	***1/147 (0*.*7%)***	***14/147 (9*.*5%)***
***Asia & Africa***	***LT-only***	***333***	***0/333 (0%)***	***1/333 (0*.*3%)***	***1/333 (0*.*3%)***	***0/333 (0%)***	***0/333 (0%)***	***93/333 (27*.*9%)***	***63/333 (18*.*9%)***	***1/333 (0*.*3%)***	***29/333 (8*.*7%)***
	***ST-only***	***146***	***26/146 (17*.*8%)***	***1/146 (0*.*7%)***	***0/146 (0%)***	***0/146 (0%)***	***1/146 (0*.*7%)***	***48/146 (32*.*9%)***	***26/146 (17*.*8%)***	***2/146 (1*.*4%)***	***20/146 (13*.*7%)***
	***LT/ST***	***232***	***0/232 (0%)***	***56/232 (24*.*1%)***	***13/232 (5*.*6%)***	***12/232 (5*.*2%)***	***31/232 (13*.*4%)***	***39/232 (16*.*8%)***	***14/232 (6*.*0%)***	***1/232 (0*.*4%)***	***24/232 (10*.*3%)***
	***ST+LT/ST***	***378***	***26/378 (6*.*9%)***	***57/378 (15*.*1%)***	***13/378 (3*.*4%)***	***12/378 (3*.*2%)***	***32/378 (8*.*5%)***	***8/378 (23*.*0%)***	***40/378 (10*.*6%)***	***3/378 (0*.*8%)***	***44/378 (11*.*6%)***

^**a**^ CFA/II strains are defined as encoding CS3 either alone or in combination with either CS1 or CS2 but never both CS1 and CS2. Very rarely isolates that encode CS1 without CS3 have been reported,[26] but the rare CFs of this nature recovered in GEMS are not included in this table.

^**b**^ CFA/IV strains are defined as encoding CS6 either alone or in combination with either CS4 or CS5, but never both CS4 and CS5. Very rarely isolates that encode CS5 without CS6 have been reported but the few such isolates recovered in GEMS are not included in this table.

### Minor CFs among ETEC case isolates lacking major CFs

Recognizing that 34.0% of ST-only and LT/ST strains and 82.0% of LT-only strains do not encode a major CF, we investigated those isolates to detect ones that encode exclusively one of the following characterized minor CF antigens: CS7, CS12, CS13, CS14, CS17, CS18, CS19, CS20, CS21 or CS30. We determined the proportion of ETEC MSD cases that had isolates encoding one of these minor CFs in the absence of a major CF and that accounted for at least 5.0% of the overall case isolates of that toxin genotype (**Table 5**).

**Table 5:** Major and minor colonization factors among ETEC isolates of different toxin genotypes, all sites combined.

	Case isolates (N = 806)	Isolates with major CFs (CFA/I, CS1-CS6)	Isolates without major CFs	CS7-only^a^	CS12-only^a^	CS13-only^a^	CS14-only^a^	CS17-only^a^	CS18-only^a^	CS19-only^a^	CS20-only^a^	CS21-only^a^	CS30-only^a^	Isolates with major or minor CFs
LT-only, individual CS-only	256	64 (25.0%)^b^	192 (82.0%)^b^	20 (7.8%)^b^	5 (2.0%)^b^	4 (1.6%)^c^	11 (4.3%)^b^	17 (6.6%)^b^	4 (1.6%)^c^	3 (1.3%)^c^^1^	8 (3.2%)^c^	4 (1.6%)^b^	-	140 (54.7%)^b^
ST-only, individual CS-only	291	187 (64.3%)^d^	104 (35.7%)^d^	0 (0%)^d^	0 (0%)^d^	0 (0%)^e^	58 (19.9%)^d^	1 (0.3%)^d^	0 (0%)^e^	0 (0%)^e1^	0 (0%)^e^	5 (1.7%)^d^	-	251 (86.3%)^d^
LT/ST, individual CS-only	259	176 (68.0%)^f^	83 (32.0%)^f^	1 (0.4%)^f^	9 (3.5%)^f^	2 (0.8%)^g^	4 (1.5%)^f^	0 (0%)^f^	1 (0.4%)^g^	0 (0%)^g1^	7 (2.7%)^g^	2 (0.8%)^f^	5 (1.9%)^g^	207 (79.9%)^f^
ST-only + LT/ST, individual CS-only^a^	550	363 (66.0%)^h^	187 (34.0%)^h^	1 (0.2%)^h^	9 (1.6%)^h^	2 (0.4%)^i^	62 (11.3%)^h^	1 (0.2%)^h^	1 (0.2%)^i^	0 (0%)^i1^	7 (1.3%)^i^	7 (1.3%)^h^	-	458 (83.3%)^h^

^a^ These strains express only the indicated minor CF in the absence of either major CF antigens or other minor CF antigens

^b^ Percent of all 256 LT-only strains

^c^ Percent of 250 LT-only strains (6 strains were not recoverable from -70^o^ C storage for testing)

^c1^ Percent of 225 LT-only strains

^d^ Percent of all 291 ST-only strains

^e^ Percent of 290 ST-only strains (1 strain was not recoverable from -70^o^ C storage for testing)

^e1^ Percent of 266 ST-only strains

^f^ Percent of all 259 LT/ST strains

^g^ Percent of 257 LT/ST strains (2 strains were not recoverable from -70^o^ C storage for testing)

^g1^ Percent of 236 LT/ST strains

^h^ Percent of all 550 ST-only plus LT/ST strains

^i^ Percent of 547 ST-only plus LT/ST strains (3 strains were not recoverable from -70^o^ C storage for testing)

^i1^ Percent of 502 ST-only plus LT/ST strains

Among MSD cases with ST-only ETEC, only CS14, identified in 58 ST-only cases (19.9%), reached a prevalence of ≥5% (**Table 5**); four MSD cases with LT/ST isolates lacking major CFs also encoded solely CS14 (1.5%). Cases isolates having other minor CS antigens encoded as the sole CS were uncommon (<5%) among ST-only and LT/ST isolates. As an example, we cite recently described CS30 [19]. Among the 83 LT/ST cases whose isolates lacked major CFs, 16, all LT/STp genotype, encoded CS30 but only five cases had CS30 as the sole CS. The 192 MSD cases with LT-only isolates lacking major CFs included strains encoding CS7 (7.8%) or CS17 (6.6%) as sole CS antigens, yielding a cumulative prevalence of 14.4% for LT-only strains encoding one of those two minor CFs (**Table 5**).

### Conditional logistic regression analyses to assess the strength of association between CF-toxin genotypes and MSD

The GEMS case/control design allowed us to assess the strength of association between the various major and minor CFs and MSD among cases versus their matched controls using conditional logistic regression models. To document the validity of this methodology, we first quantified the strength of association with MSD of the major CFs (**Table 6**), since they are widely regarded as true virulence attributes. ST-only and LT/ST strains encoding CFA/I, CFA/II and CFA/IV were all significantly associated with MSD (p<0.0001, p = 0.006, p<0.0001, respectively). In contrast, LT-only strains encoding only CS6 or CS5 and CS6 were not significantly associated with MSD (p>0.05; **Table 6**).

**Table 6:** Odds ratios for association with moderate-to-severe diarrhea of ETEC encoding major or minor colonization factors in combination with different toxin genotypes.

		Cases N = 9439	Controls N = 13,129	Pooled matched odds ratio (95% CI)	p Value	p value for heterogeneity^a^
Major colonization factors	Toxin type					
CFA/I	ST-only + LT/ST	112 (1.19%)	26 (0.20%)	1.85 (1.52–2.22)	<0.0001	0.97
Any CFA/II family^b1^	ST-only + LT/ST	77 (0.82%)	57 (0.43%)	1.36 (1.08–1·69)	0.006	0.97
Any CFA/IV family^b2^	ST-only + LT/ST	174 (1.84%)	87 (0.66%)	1.62 (1.39–1.88)	<0.0001	0.88
CS6-only	LT-only	43 (0.46%)	63 (0.48%)	0.95 (0.69–1.25)	0.70	0.89
CS5+CS6	LT-only	18 (0.19%)	28 (0.21%)	0.98 (0.59–1.53)	0.90	0.97
Minor colonization factors						
CS7 ^b3^^,^^c^	ST-only + LT/ST	1 (0.01%)	1 (0.008%)	1.88 (0.21–6.74)	0.40	0.64
	LT-only	20 (0.21%)	12 (0.09%)	1.49 (0.93–2.23)	0.071	0.93
CS12^b4^^,^^c^	ST-only + LT/ST	9 (0.095%)	27 (0.22%)	0.56 (0.27–1.00)	0.076	0.92
	LT-only	5 (0.053%)	5 (0.038%)	1.46 (0.55–3.05)	0.30	0.86
CS13^b5^^,^^c^	ST-only + LT/ST	2 (0.021%)	3 (0.023%)	1.40 (0.29–4.01)	0.50	0.70
	LT-only	4 (0.042%)	30 (0.22%)	0.33 (0.11–0.75)	0.021	0.47
CS14^b6^^,^^c^	ST-only + LT/ST	62 (0.66%)	33 (0.25%)	1.52 (1.17–1.94)	0.0011	0.72
	LT-only	11 (0.12%)	10 (0.08%)	1.19 (0.63–2.03)	0.50	0.57
CS17^b7^^,^^c^	ST-only + LT/ST	1 (0.011%)	2 (0.015%)	1.06 (0.12–3.82)	0.90	0.78
	LT-only	17 (0.18%)	15 (0.11%)	1.24 (0.74–1.93)	0.30	0.78
CS18^b8^^,^^c^	ST-only + LT/ST	1 (0.011%)	0 (0%)	3.03 (0.34–10.96)	0.17	
	LT-only	4 (0.042%)	2 (0.015%)	1.57 (0.52–3.49)	0.30	0.82
CS19^b9^^,^^c^	ST-only + LT/ST	0 (0%)	6 (0.046%)	0.22 (0.002–1.46)	0.28	0.81
	LT-only	3 (0.032%)	13 (0.099%)	0.51 (0.14–1.26)	0.21	0.39
CS20^b10^^,^^c^	ST-only + LT/ST	7 (0.074%)	21 (0.16%)	0.66 (0.29–1.25)	0.25	0·77
	LT-only	8 (0.085%)	13 (0.099%)	0.95 (0.44–1.75)	0.80	0.98
CS21^b11^^,^^c^	ST-only + LT/ST	7 (0.074%)	12 (0.091%)	0.93 (0.41–1.78)	0.80	0.99
	LT-only	4 (0.042%)	0 (0%)	2.84 (0.95–6.42)	0.028	0.72
CS30^b12^^,^^c^	LT/ST	5 (0.053%)	7 (0.053%)	1.13 (0.42–2.34)	0.70	0.48

^a^ There was no significant heterogeneity across GEMS sites (by chi square test for heterogeneity).

^b1^ ST and CS3-only was not detected in Bangladesh.

^b2^ ST and CS4 & SC6-only was not found Gambia, Mozambique and Bangladesh.

^b3^ ST and CS7-only was restricted to Gambia and India.

^b4^ LT and CS12-only was not found in Gambia.

^b5^ ST and CS13-only was not found in Mali, India and Pakistan.

^b6^ LT and CS14-only was not found in Mali and Bangladesh.

^b7^ ST and CS17-only was found only in Mozambique, Kenya and Bangladesh.

^b8^ ST and CS18-only was found only in Gambia; LT and CS18-only was found only in Mali and Kenya.

^b9^ ST and CS19-only was detected in Kenya, India and Bangladesh; LT and CS19-only was not found Mozambique and Kenya.

^b10^ LT and CS20-only was not detected in Bangladesh.

^b11^ LT and CS21-only was detected only in Kenya and Pakistan.

^b12^ LT-ST and CS30-only was not found in Bangladesh.

^c^ Excludes strains that co-encoded a major colonization factor such as CFA/I, CFA/II, CFA/IV, and all other minor colonization factors.

Note—These analyses are based on the entire GEMS dataset. In each conditional logistic regression model the dependent variable was MSD (cases or controls), while the independent variable was the presence (coded as 1) or absence (coded as 0) of a specified ETEC CF-toxin profile

Conditional logistic regression modeling was then performed to assess the association between LT/ST and ST-only ETEC expressing one of the 10 minor CFs alone (CS7, CS12, CS13, CS14, CS17, CS18, CS19, CS20, CS21 or CS30) and MSD. Among ST-only and LT/ST ETEC strains encoding exclusively a single minor CF but no major CF, only CS14 was significantly associated with MSD (**Table 6**).

When conditional logistic regression was performed for LT-only cases and ETEC strains encoding exclusively one of these ten minor CFs, only CS21 exhibited a significant association (p = 0.028). However, LT-only isolates expressing CS21 exclusively were uncommon among cases (N = 4) and matched controls (N = 0). CS7 did not show a significant association (p = 0.071) but the sample sizes of cases (N = 20) and controls (N = 12) were small. We did not use a Bonferroni adjustment for these individual conditional logistic regression analyses of the association of individual minor CFs with MSD, as in each instance an individual hypothesis was being tested [41–44].

### Phenotypic expression of CFs

ETEC isolates from 443 cases (338 encoding major CFs and 105 encoding a single minor CF) were tested by dot blot immunoassay with specific anti-CF antibodies to determine the percent that phenotypically expressed on their bacterial surface the encoded major CF antigens. Of ETEC encoding CFA/I or CS1-CS5, 73.8–95.1% of isolates tested were dot blot-positive (**Table 7**); the exceptions were the 65 CS6-only isolates tested that showed only 38.5% positivity. Among ETEC case isolates encoding one of the four minor CFs tested, dot blot immunoassay positivity ranged from 67.2% for CS17 to 94.4% for CS7.

**Table 7:** ETEC isolates from MSD cases encoding colonization factor (CF) genes detected by PCR and phenotypic expression as detected by dot blot immunoassay using CF-specific antibodies.

Colonization factor	Total number of GEMS strains positive for this CF genotype	Total number of strains tested by dot blot immunoassay	Phenotypic expression of CF among PCR-positive isolates^a^ (%)	
Major CFs				
CFA/I				
			*With anti-CFA/I*^*b*^	
	113	81	77/81 (95.1%)	
CFA/II				
			*With anti-CS1*^b^	*With anti-CS3* ^b^
CS1 & CS3	23	19	18/19 (94.7%)	18/19 (94.7%)
			*With anti-CS2* ^b^	*With anti-CS3* ^b^
CS2 & CS3	39	30	27/30 (90.0%)	27/30 (90.0%)
			-	*With anti-CS3*
CS3-only	17	15	-	14/15 (93.3%) ^b^
CFA/IV				
			*With anti-CS4* ^b^	*With anti-CS6* ^b^
CS4 & CS6	8	6	5/6 (83.3%)	5/6 (83.3%)
			*With anti-CS5* ^b^	*With anti-CS6* ^b^
CS5 & CS6	146	122	90/122 (73.8%)	90/122 (73.8%)
			-	*With anti-CS6* ^b^
CS6-only	81	65	-	25/65 (38.5%)
Minor CFs				
CS7^c^	21	18	17/18 (94.4%)	
CS12^c^	14	12	9/12 (75.0%)	
CS14^c^	73	61	41/61 (67.2%)	
CS17^c^	18	14	12/14 (85.7%)	

^a^ Number of isolates positive by dot blot immunoassay/total number of PCR-positive isolates tested

^b^ Antibody used in the dot blot immunoassay

^c^ Negative for all other colonization factors. Dot blot immunoassays were performed with the homologous antiserum

## Discussion

The GEMS case/control study demonstrated that ST-only and LT/ST ETEC, the enterotoxin genotypes exhibited by circa two-thirds of ETEC isolates from patients, were strongly associated with MSD [1,49]. Field studies involving small pediatric cohorts prospectively followed under active household surveillance document that these toxin types are also incriminated as causing milder diarrhea [13,50,51]. Most LT-only strains are not associated with diarrhea [1,13,50,52,53], as some descend from LT/ST strains through loss of genes encoding ST and a CF [3]. Nevertheless, evidence from diarrhea outbreaks in industrialized countries [54], experimental challenges in U.S. volunteers [12,55], and epidemiological studies in developing countries indicate that a subset of LT-only strains do appear to be *bona fide* diarrheal pathogens [13,14], and it would be desirable to prevent diarrhea caused by that subset. The quandary, heretofore, has been how to identify accessory virulence attributes that distinguish the subset of LT-only ETEC that can cause diarrhea versus the non-pathogenic LT-only strains. Since individual *E*. *coli* colonies from each GEMS MSD case and their controls were tested for genes encoding LT and ST, it was possible to examine what major and minor CFs were encoded by the GEMS LT-only isolates as well as by isolates of the other toxin genotypes.

Four cardinal findings emerged from examining the CF genotypes of the GEMS ETEC isolates. First, GEMS results confirm that ETEC vaccines based on stimulating immune responses to the major CFs (CFA/I and CS1-6), if highly efficacious in blocking CF-mediated attachment to enterocytes, could prevent diarrhea caused by up to 66% of the ST-only and LT/ST strains, the toxin genotypes strongly incriminated as pathogens (**Table 5**). The fact that ETEC encoding these CFs were observed in a very large study involving multiple representative sites in sub-Saharan Africa and South Asia validates that ETEC vaccine strategy for the geographic regions where 80% of young child diarrheal deaths occur worldwide. In contrast, major CFs were uncommon among LT-only isolates.

Important avenues of ETEC vaccinology research have focused on identifying additional CFs among ST-only and LT/ST isolates that lack CFA/I, CFA/II and CFA/IV and to identify minor CFs that might be targets for protective immune responses directed against the subset of LT-only strains that are pathogenic. Thus, the second cardinal observation is identification of the proportion of strains in each toxin genotype that lacked a major CF but that exclusively expressed one minor CF, including either CS7, CS12, CS13, CS14, CS17, CS18, CS19, CS20, CS21 or CS30. Collectively these minor CFs raised the percent of ST-only cases having a recognized CF from 64.3% (187/291) to 86.3% (251/291) and raised the percent of LT/ST cases having a recognized CF from 68.0% (176/259) to 79.9% (207/259) (**Table 5**). The percent of MSD cases with LT-only ETEC having a recognized CF similarly rose from 25.0% (64/256) to 54.7% (140/256).

Although minor CFs encoded by the different toxin genotypes of ETEC strains collectively raised the proportion of cases that had a CF target, it was not known if these minor CFs also identified these strains as being pathogenic, i.e., significantly associated with MSD, as were the major CFs. Moreover, most individual minor CFs were uncommon, defined as <5% of strains of a toxin genotype that lacked a major CF. Thus, a third cardinal observation was assessment of the strength of association between MSD and ETEC encoding the various major and minor CFs within the different toxin genotypes. These novel analyses (**Table 6**) revealed that the major CF families encoded by ST-only or LT/ST strains are significantly associated with MSD (CFA/I and CFA/IV, p<0.0001; CFA/II, p = 0.006). Thus, CFA/I, CFA/II and CFA/IV are not only surface-exposed targets for effector immune responses, but when expressed by LT/ST and ST-only ETEC they are markers indicating that these strains are strongly incriminated as diarrheal pathogens. In contrast, LT-only isolates encoding CS6 alone or CS5 and CS6 were not significantly associated with MSD (**Table 6**), nor was there a trend.

A notable proportion (19.9%) of ST-only isolates encoded CS14 alone, i.e., with no other minor or major CFs, and these were significantly associated with MSD (p = 0.001) (**Table 6**). No other individual minor CF was both common and significantly associated with MSD among the cases infected with ST-only and LT/ST isolates.

Whereas LT-only strains encoding exclusively CS21 were significantly associated with MSD (p = 0.021), these strains were distinctly uncommon. LT-only strains encoding exclusively CS7 were prevalent (7.8%, Table 5) but they were not significantly associated with MSD (p = 0.07). However, the sample sizes of cases (N = 20) and controls (N = 12) with LT-only encoding exclusively CS7 were small, so further investigation of this CF should be encouraged to explore the potential role of CS7 for potential inclusion in an ETEC vaccine. Support for this notion comes from two small infant cohort studies in Guinea-Bissau (N = 200) and Egypt (N = 348) that assessed the association of ETEC encoding specific CFs with diarrhea using logistic regression models and reported significant associations of LT-only CS7 with infant diarrhea [13,14].

Since experimental challenge with an LT-only strain encoding CS17 fomented diarrhea in adult volunteers [12], it was somewhat surprising that LT-only/CS17-only strains were not significantly associated with MSD in young children in GEMS. Nevertheless, because CS17 shares epitopes with CFA/I, CS1 and CS2, and CS7 shares epitopes with CS5, the immune responses to these major CFs within a vaccine that also contains a LT toxoid to stimulate anti-LT could collectively confer protection against pathogenic LT-only strains encoding CS17 and CS7 [56].

The fourth key observation is that inclusion of CS14 expands the breadth of vaccine coverage against ST-only pathogens, raising it from 64.3% to 84.2% (**Table 6**). Obviously, antigenic expansion, particularly if multiple minor CFs beyond CS14 (e.g., CS7, CS17, CS21) were to be added to a major CF-based ETEC vaccine, would increase the vaccine’s complexity and cost. Nevertheless, there is precedent for successfully addressing this problem with other bacterial vaccines. Pneumococcal conjugate vaccines were expanded from 7-valent to 13-valent to allow broader global coverage, while multivalent meningococcal conjugate vaccines currently include four separate serogroup conjugates. Some ETEC vaccine strategies, such as attenuated *Shigella* live vectors encoding two separate CFs per vector strain, can be adapted relatively easily to express additional CFs [57].

Another approach to broaden coverage of an ETEC vaccine is based on formulating a mix of fimbrial tip adhesin proteins [5]. Fimbrial CFs can be classified based on the amino acid sequence relatedness of their tip adhesin proteins, with several important ETEC CFs falling into Class 5 fimbriae assembled by the alternate chaperone pathway. Whereas the major fimbrial subunit proteins that create the stalks of these fimbriae differ substantially from one another antigenically, their tip adhesin proteins are highly conserved into three sub-classes [58,59]. Antibody against one adhesin of the subclass cross protects against attachment by other members. Thus, protection may also be broadened by this strategy. Selecting which tip adhesins to include in a multivalent vaccine requires knowing the frequency of the CFs among ETEC globally; so the GEMS data inform this vaccine strategy as well. Another strategy to broaden ETEC vaccine coverage would include non-fimbrial surface antigens, e.g., EtpA (a non-fimbrial adhesin) and EatA (a serine protease) [60].

Among ETEC strains encoding a major CF other than CS6 alone or a minor CF, 67.2% - 95.1% of isolates reacted with the specific homologous anti-CF antibody by dot blot immunoassay, thereby documenting phenotypic expression. The exceptions were the CS6-only isolates of which only 25/65 (38.5%) were dot blot-positive (**Table 7**) whether they were LT-only isolates encoding CS6-only (10/32, 31.3%) or ST-encoding CS6-only strains (15/33, 45.5%). The expression of CFs is highly regulated [61–63], with temperature, bile, concentrations of glucose, glutamine and iron, and proximity to epithelial cells all influencing expression [64–67]. Thus, one explanation for lack of expression is that the *in vitro* growth conditions that we utilized did not induce the regulated biosynthesis of isolates encoding certain CFs. Transcriptional regulators such as CfaD (also called CfaR) and Rns that are members of the AraC family of transcriptional regulators modulate the expression of CFA/I, CS1, CS2, CS4 and CS5 [61,62,66,68]. In contrast, although certain growth conditions such as temperature modify CS6 expression [67], no specific positive regulator has been identified for CS6 [68–70]. Alternatively, isolates that are PCR-positive but dot blot-negative may have single nucleotide polymorphisms, minor mutations in structural or chaperone genes or lower copy number plasmids that still allow amplification by PCR but may diminish or abrogate expression of the CF [69,71].

One theoretical limitation of our study is that the PCR primers designed to detect ST at the field sites were optimized for STh; thus some STp-only isolates may have been missed. LT-STp strains from cases were not under-estimated in GEMS because all LT-only strains were re-tested with PCRs individually optimized for STp and STh in the Chilean Reference Laboratory and upon re-testing only a limited number of LT-only isolates were found to be LT/STp. We believe that few cases and controls with STp-only were missed. A GEMS follow-on study detected STp and STh in genomic DNA extracted from whole stool specimens of a subset of 5304 case/matched control pairs using a TaqMan Card-based quantitative real-time PCR (qPCR) methodology and documented that the ST burden was overwhelmingly attributed to STh [49]. Optimized detection of STp by qPCR increased the overall ETEC disease burden estimate by only 15% versus what was recorded using the gel-based PCR methodology at the field sites [49]. This is similar to the overall difference based on presumed gene loss between primary isolation and results of re-testing strains following storage and transport to the Reference Laboratory. Other studies have found that STp-only isolates are uncommon compared to STh-only when methods sensitive for STp are used [13].

Analyzing the array of CFs among GEMS ETEC isolates has provided important information to guide ETEC vaccine development and future deployment. Since ST-only and LT/ST strains are strongly incriminated as the key ETEC pathogens, a fimbrial-based ETEC vaccine that included CFA/I, CS1-6 and CS14, if highly efficacious, could theoretically confer protection against up to ~77% of such ETEC pathogens.

## Supporting information

S1 Checklist STROBE checklist. (DOC)

S1 File (XLSX)

## References

[1] Kotloff KL, Nataro JP, Blackwelder WC, Nasrin D, Farag TH, Panchalingam S, et al Burden and aetiology of diarrhoeal disease in infants and young children in developing countries (the Global Enteric Multicenter Study, GEMS): a prospective, case-control study. Lancet. 2013; 382(9888):209–22. 10.1016/S0140-6736(13)60844-2 23680352

[2] Hyams KC, Bourgeois AL, Merrell BR, Rozmahel R, Escamilla J, Thornton SA, et al Diarrheal disease during Operation Desert Shield. N Engl J Med. 1991; 325:1423–8. 10.1056/NEJM199111143252006 1656260

[3] Levine MM. *Escherichia coli* that cause diarrhea: enterotoxigenic, enteropathogenic, enteroinvasive, enterohemorrhagic, and enteroadherent. J Infect Dis. 1987; 155:377–89. 3543152 10.1093/infdis/155.3.377

[4] Levine MM, Ristaino P, Marley G, Smyth C, Knutton S, Boedeker E, et al Coli surface antigens 1 and 3 of colonization factor antigen II- positive enterotoxigenic *Escherichia coli*: morphology, purification, and immune responses in humans. Infect Immun. 1984; 44:409–20. 6370866 10.1128/iai.44.2.409-420.1984 PMC263534

[5] Sincock SA, Hall ER, Woods CM, O'Dowd A, Poole ST, McVeigh AL, et al Immunogenicity of a prototype enterotoxigenic *Escherichia coli* adhesin vaccine in mice and nonhuman primates. Vaccine. 2016; 34(2):284–91. 10.1016/j.vaccine.2015.11.017 26597148

[6] Lundgren A, Bourgeois L, Carlin N, Clements J, Gustafsson B, Hartford M, et al Safety and immunogenicity of an improved oral inactivated multivalent enterotoxigenic *Escherichia coli* (ETEC) vaccine administered alone and together with dmLT adjuvant in a double-blind, randomized, placebo-controlled Phase I study. Vaccine. 2014; 32(52):7077–84. 10.1016/j.vaccine.2014.10.069 25444830

[7] Turner AK, Stephens JC, Beavis JC, Greenwood J, Gewert C, Randall R, et al Generation and characterization of a live attenuated enterotoxigenic *Escherichia coli* combination vaccine expressing six colonization factors and heat-labile toxin subunit B. Clin Vaccine Immunol. 2011; 18(12):2128–35. 10.1128/CVI.05345-11 21994355 PMC3232708

[8] Barry EM, Altboum Z, Losonsky G, Levine MM. Immune responses elicited against multiple enterotoxigenic *Escherichia coli* fimbriae and mutant LT expressed in attenuated *Shigella* vaccine strains. Vaccine. 2003; 21(5–6):333–40. 12531629 10.1016/s0264-410x(02)00611-4

[9] Duan Q, Lu T, Garcia C, Yanez C, Nandre RM, Sack DA, et al Co-administered Tag-Less Toxoid Fusion 3xSTaN12S-mnLTR192G/L211A and CFA/I/II/IV MEFA (Multiepitope Fusion Antigen) Induce Neutralizing Antibodies to 7 Adhesins (CFA/I, CS1-CS6) and Both Enterotoxins (LT, STa) of Enterotoxigenic Escherichia coli (ETEC). Front Microbiol. 2018; 9:1198 10.3389/fmicb.2018.01198 eCollection;%2018.:1198. 29922268 PMC5996201

[10] Taxt AM, Diaz Y, Aasland R, Clements JD, Nataro JP, Sommerfelt H, et al Towards Rational Design of a Toxoid Vaccine against the Heat-Stable Toxin of *Escherichia coli*. Infect Immun. 2016; 84(4):1239–49. 10.1128/IAI.01225-15 26883587 PMC4807477

[11] Robbins JB, Schneerson R, Szu SC. Perspective: hypothesis: serum IgG antibody is sufficient to confer protection against infectious diseases by inactivating the inoculum. J Infect Dis. 1995; 171(6):1387–98. 7769272 10.1093/infdis/171.6.1387

[12] McKenzie R, Porter CK, Cantrell JA, Denearing B, O'Dowd A, Grahek SL, et al Volunteer challenge with enterotoxigenic *Escherichia coli* that express intestinal colonization factor fimbriae CS17 and CS19. J Infect Dis. 2011; 204(1):60–4. 10.1093/infdis/jir220 21628659 PMC3105037

[13] Steinsland H, Valentiner-Branth P, Perch M, Dias F, Fischer TK, Aaby P, et al Enterotoxigenic *Escherichia coli* infections and diarrhea in a cohort of young children in Guinea-Bissau. J Infect Dis. 2002; 186(12):1740–7. 10.1086/345817 12447759

[14] Mansour A, Shaheen HI, Amine M, Hassan K, Sanders JW, Riddle MS, et al Pathogenicity and phenotypic characterization of enterotoxigenic *Escherichia coli* isolates from a birth cohort of children in rural Egypt. J Clin Microbiol. 2014; 52(2):587–91. 10.1128/JCM.01639-13 24478492 PMC3911313

[15] Gaastra W, Svennerholm AM. Colonization factors of human enterotoxigenic *Escherichia coli* (ETEC). Trends Microbiol. 1996; 4(11):444–52. 8950814 10.1016/0966-842x(96)10068-8

[16] del Canto F., Valenzuela P, Cantero L, Bronstein J, Blanco JE, Blanco J, et al Distribution of classical and nonclassical virulence genes in enterotoxigenic *Escherichia coli* isolates from Chilean children and tRNA gene screening for putative insertion sites for genomic islands. J Clin Microbiol. 2011; 49(9):3198–203. 10.1128/JCM.02473-10 21775541 PMC3165568

[17] del Canto F., O'Ryan M, Pardo M, Torres A, Gutierrez D, Cadiz L, et al Chaperone-Usher Pili Loci of Colonization Factor-Negative Human Enterotoxigenic *Escherichia coli*. Front Cell Infect Microbiol. 2017; 6:200 10.3389/fcimb.2016.00200 eCollection;%2016.:200. 28111618 PMC5216030

[18] del Canto F., Botkin DJ, Valenzuela P, Popov V, Ruiz-Perez F, Nataro JP, et al Identification of Coli Surface Antigen 23, a novel adhesin of enterotoxigenic *Escherichia coli*. Infect Immun. 2012; 80(8):2791–801. 10.1128/IAI.00263-12 22645287 PMC3434557

[19] von Mentzer A, Tobias J, Wiklund G, Nordqvist S, Aslett M, Dougan G, et al Identification and characterization of the novel colonization factor CS30 based on whole genome sequencing in enterotoxigenic *Escherichia coli* (ETEC). Sci Rep. 2017; 7(1):12514–743. 10.1038/s41598-017-12743-3 28970563 PMC5624918

[20] Levine MM, Kotloff KL, Nataro JP, Muhsen K. The Global Enteric Multicenter Study (GEMS): Impetus, Rationale, and Genesis. Clin Infect Dis. 2012; 55 Suppl 4:S215–24. 10.1093/cid/cis761.:S215-S224. 23169934 PMC3502311

[21] Farag TH, Nasrin D, Wu Y, Muhsen K, Blackwelder WC, Sommerfelt H, et al Some Epidemiologic, Clinical, Microbiologic, and Organizational Assumptions That Influenced the Design and Performance of the Global Enteric Multicenter Study (GEMS). Clin Infect Dis. 2012; 55 Suppl 4:S225–31. 10.1093/cid/cis787.:S225-S231 23169935 PMC3502315

[22] Panchalingam S, Antonio M, Hossain A, Mandomando I, Ochieng B, Oundo J, et al Diagnostic Microbiologic Methods in the GEMS-1 Case/Control Study. Clin Infect Dis. 2012; 55 Suppl 4:S294–302. 10.1093/cid/cis754.:S294-S302 23169941 PMC3502308

[23] Kotloff KL, Blackwelder WC, Nasrin D, Nataro JP, Farag TH, van EA, et al The Global Enteric Multicenter Study (GEMS) of Diarrheal Disease in Infants and Young Children in Developing Countries: Epidemiologic and Clinical Methods of the Case/Control Study. Clin Infect Dis. 2012; 55 Suppl 4:S232–45. 10.1093/cid/cis753.:S232-S245 23169936 PMC3502307

[24] Vidal R, Vidal M, Lagos R, Levine M, Prado V. Multiplex PCR for diagnosis of enteric infections associated with diarrheagenic *Escherichia coli*. J Clin Microbiol. 2004; 42(4):1787–9. 10.1128/JCM.42.4.1787-1789.2004 15071051 PMC387562

[25] Rodas C, Iniguez V, Qadri F, Wiklund G, Svennerholm AM, Sjoling A. Development of multiplex PCR assays for detection of enterotoxigenic *Escherichia coli* colonization factors and toxins. J Clin Microbiol. 2009; 47(4):1218–20. 10.1128/JCM.00316-09 19244463 PMC2668350

[26] Vidal RM, Valenzuela P, Baker K, Lagos R, Esparza M, Livio S, et al Characterization of the most prevalent colonization factor antigens present in Chilean clinical enterotoxigenic *Escherichia coli* strains using a new multiplex polymerase chain reaction. Diagn Microbiol Infect Dis. 2009; 65(3):217–23. 10.1016/j.diagmicrobio.2009.07.005 19733027

[27] Sjoling A, Wiklund G, Savarino SJ, Cohen DI, Svennerholm AM. Comparative analyses of phenotypic and genotypic methods for detection of enterotoxigenic *Escherichia coli* toxins and colonization factors. J Clin Microbiol. 2007; 45(10):3295–301. 10.1128/JCM.00471-07 17687011 PMC2045327

[28] Pichel MG, Binsztein N, Qadri F, Giron JA. Type IV longus pilus of enterotoxigenic *Escherichia coli*: occurrence and association with toxin types and colonization factors among strains isolated in Argentina. J Clin Microbiol. 2002; 40(2):694–7. 10.1128/JCM.40.2.694-697.2002 11826000 PMC153402

[29] McKenzie R, Porter CK, Cantrell JA, Denearing B, O'Dowd A, Grahek SL, et al Volunteer challenge with enterotoxigenic *Escherichia coli* that express intestinal colonization factor fimbriae CS17 and CS19. J Infect Dis. 2011; 204(1):60–4. 10.1093/infdis/jir220 21628659 PMC3105037

[30] Qadri F, Svennerholm AM, Faruque AS, Sack RB. Enterotoxigenic *Escherichia coli* in developing countries: epidemiology, microbiology, clinical features, treatment, and prevention. Clin Microbiol Rev. 2005; 18(3):465–83. 10.1128/CMR.18.3.465-483.2005 16020685 PMC1195967

[31] Gonzales L, Sanchez S, Zambrana S, Iniguez V, Wiklund G, Svennerholm AM, et al Molecular characterization of enterotoxigenic Escherichia coli isolates recovered from children with diarrhea during a 4-year period (2007 to 2010) in Bolivia. J Clin Microbiol. 2013; 51(4):1219–25. 10.1128/JCM.02971-12 23390275 PMC3666779

[32] Tacket CO, Maneval DR, Levine MM. Purification, morphology, and genetics of a new fimbrial putative colonization factor of enterotoxigenic *Escherichia coli* O159:H4. Infect Immun. 1987; 55:1063–9. 2883122 10.1128/iai.55.5.1063-1069.1987 PMC260469

[33] Giron JA, Levine MM, Kaper JB. Longus: a long pilus ultrastructure produced by human enterotoxigenic *Escherichia coli*. Mol Microbiol. 1994; 12(1):71–82. 7914665 10.1111/j.1365-2958.1994.tb00996.x

[34] Giron JA, Viboud GI, Sperandio V, Gomez-Duarte OG, Maneval DR, Albert MJ, et al Prevalence and association of the longus pilus structural gene (lngA) with colonization factor antigens, enterotoxin types, and serotypes of enterotoxigenic *Escherichia coli*. Infect Immun. 1995; 63:4195–8. 7558343 10.1128/iai.63.10.4195-4198.1995 PMC173594

[35] Guevara CP, Luiz WB, Sierra A, Cruz C, Qadri F, Kaushik RS, et al Enterotoxigenic *Escherichia coli* CS21 pilus contributes to adhesion to intestinal cells and to pathogenesis under in vivo conditions. Microbiology. 2013; 159(Pt 8):1725–35. 10.1099/mic.0.065532-0 23760820 PMC3749052

[36] Nuccio SP, Baumler AJ. Evolution of the chaperone/usher assembly pathway: fimbrial classification goes Greek. Microbiol Mol Biol Rev. 2007; 71(4):551–75. 10.1128/MMBR.00014-07 18063717 PMC2168650

[37] Madhavan TP, Sakellaris H. Colonization factors of enterotoxigenic *Escherichia coli*. Adv Appl Microbiol. 2015; 90:155–97. 10.1016/bs.aambs.2014.09.003 Epub;%2014 Nov 12.:155–97. 25596032

[38] Isidean SD, Riddle MS, Savarino SJ, Porter CK. A systematic review of ETEC epidemiology focusing on colonization factor and toxin expression. Vaccine. 2011; 29(37):6167–78. 10.1016/j.vaccine.2011.06.084 21723899

[39] Blackwelder WC, Biswas K, Wu Y, Kotloff KL, Farag TH, Nasrin D, et al Statistical Methods in the Global Enteric Multicenter Study (GEMS). Clin Infect Dis. 2012; 55 Suppl 4:S246–53. 10.1093/cid/cis788.:S246-S253 23169937 PMC3502316

[40] Firth D. Bias reduction of maximum likelihood estimates. Biometrika. 1993; 80:27–38.

[41] Rothman KJ. No adjustments are needed for multiple comparisons. Epidemiology. 1990; 1(1):43–6. 2081237

[42] Savitz DA, Olshan AF. Multiple comparisons and related issues in the interpretation of epidemiologic data. Am J Epidemiol. 1995; 142(9):904–8. 7572970 10.1093/oxfordjournals.aje.a117737

[43] Savitz DA, Olshan AF. Describing data requires no adjustment for multiple comparisons: a reply from Savitz and Olshan. Am J Epidemiol. 1998; 147(9):813–4. 9583710 10.1093/oxfordjournals.aje.a009532

[44] Perneger TV. What's wrong with Bonferroni adjustments. BMJ. 1998; 316(7139):1236–8. 9553006 10.1136/bmj.316.7139.1236 PMC1112991

[45] Evans DJ Jr., Evans DG, DuPont HL, Orskov F, Orskov. Patterns of loss of enterotoxigenicity by *Escherichia coli* isolated from adults with diarrhea: suggestive evidence for an interrelationship with serotype. Infect Immun. 1977; 17(1):105–11. 328392 10.1128/iai.17.1.105-111.1977 PMC421088

[46] Tobias J, von MA, Loayza FP, Aslett M, Page AJ, Sjoling A, et al Stability of the Encoding Plasmids and Surface Expression of CS6 Differs in Enterotoxigenic *Escherichia coli* (ETEC) Encoding Different Heat-Stable (ST) Enterotoxins (STh and STp). PLoS ONE. 2016; 11(4):e0152899 10.1371/journal.pone.0152899 27054573 PMC4824445

[47] Echeverria P, Seriwatana J, Taylor DN, Changchawalit S, Smyth CJ, Twohig J, et al Plasmids coding for colonization factor antigens I and II, heat-labile enterotoxin, and heat-stable enterotoxin A2 in *Escherichia coli*. Infect Immun. 1986; 51(2):626–30. 3510984 10.1128/iai.51.2.626-630.1986 PMC262392

[48] Evans DG, Evans DJ Jr. New surface-associated heat-labile colonization factor antigen (CFA/II) produced by enterotoxigenic Escherichia coli of serogroups O6 and O8. Infect Immun. 1978; 21(2):638–47. 80383 10.1128/iai.21.2.638-647.1978 PMC422040

[49] Liu J, Platts-Mills JA, Juma J, Kabir F, Nkeze J, Okoi C, et al Use of quantitative molecular diagnostic methods to identify causes of diarrhoea in children: a reanalysis of the GEMS case-control study. Lancet. 2016; 388(10051):1291–301. 10.1016/S0140-6736(16)31529-X 27673470 PMC5471845

[50] Levine MM, Ferreccio C, Prado V, Cayazzo M, Abrego P, Martinez J, et al Epidemiologic studies of *Escherichia coli* infections in a low socioeconomic level periurban community in Santiago, Chile. Am J Epidemiol. 1993; 138:849–69. 8237973 10.1093/oxfordjournals.aje.a116788

[51] Platts-Mills JA, Babji S, Bodhidatta L, Gratz J, Haque R, Havt A, et al Pathogen-specific burdens of community diarrhoea in developing countries: a multisite birth cohort study (MAL-ED). Lancet Glob Health. 2015; (15):10-109X. 10.1016/S2214-109X(15)00151-5 PMC7328884 26202075

[52] Satterwhite TK, Evans DG, DuPont HL, Evans DJ Jr., Role of *Escherichia coli* colonisation factor antigen in acute diarrhoea. Lancet. 1978; 2(8082):181–4. 78384 10.1016/s0140-6736(78)91921-9

[53] Viboud GI, Jouve MJ, Binsztein N, Vergara M, Rivas M, Quiroga M, et al Prospective cohort study of enterotoxigenic *Escherichia coli* infections in Argentinean children. J Clin Microbiol. 1999; 37(9):2829–33. 10449460 10.1128/jcm.37.9.2829-2833.1999 PMC85388

[54] Lumish RM, Ryder RW, Anderson DC, Wells JG, Puhr ND. Heat-labile enterotoxigenic *Escherichia coli* induced diarrhea aboard a Miami-based cruise ship. Am J Epidemiol. 1980; 111(4):432–6. 6990749 10.1093/oxfordjournals.aje.a112918

[55] Levine MM, Nalin DR, Hoover DL, Bergquist EJ, Hornick RB, Young CR. Immunity to enterotoxigenic *Escherichia coli*. Infect Immun. 1979; 23:729–36. 378834 10.1128/iai.23.3.729-736.1979 PMC414227

[56] Leach S, Lundgren A, Carlin N, Lofstrand M, Svennerholm AM. Cross-reactivity and avidity of antibody responses induced in humans by the oral inactivated multivalent enterotoxigenic *Escherichia coli* (ETEC) vaccine ETVAX. Vaccine. 2017; (17):10. 28625524 10.1016/j.vaccine.2017.06.006

[57] Barry EM, Wang J, Wu T, Davis T, Levine MM. Immunogenicity of multivalent *Shigella*-ETEC candidate vaccine strains in a guinea pig model. Vaccine. 2006; 24(18):3727–34. 10.1016/j.vaccine.2005.07.013 16169130

[58] Anantha RP, McVeigh AL, Lee LH, Agnew MK, Cassels FJ, Scott DA, et al Evolutionary and functional relationships of colonization factor antigen i and other class 5 adhesive fimbriae of enterotoxigenic *Escherichia coli*. Infect Immun. 2004; 72(12):7190–201. 10.1128/IAI.72.12.7190-7201.2004 15557644 PMC529125

[59] Gaastra W, Sommerfelt H, van DL, Kusters JG, Svennerholm AM, Grewal HM. Antigenic variation within the subunit protein of members of the colonization factor antigen I group of fimbrial proteins in human enterotoxigenic *Escherichia coli*. Int J Med Microbiol. 2002; 292(1):43–50. 12139428 10.1078/1438-4221-00189

[60] Fleckenstein J, Sheikh A, Qadri F. Novel antigens for enterotoxigenic *Escherichia coli* vaccines. Expert Rev Vaccines. 2014; 13(5):631–9. 10.1586/14760584.2014.905745 24702311 PMC4199203

[61] Caron J, Scott J. A *rns*-like regulator gene in CFA/I that controls expression of CFA/I pilin. Infect Immun. 1990; 58:874–8. 1969396 10.1128/iai.58.4.874-878.1990 PMC258554

[62] Caron J, Coffield LM, Scott JR. A plasmid-encoded regulatory gene, rns, required for expression of the CS1 and CS2 adhesins of enterotoxigenic Escherichia-coli. Proc Natl Acad Sci USA. 1989; 86:963–7. 2563591 10.1073/pnas.86.3.963 PMC286599

[63] Bodero MD, Munson GP. The Virulence Regulator Rns Activates the Expression of CS14 Pili. Genes (Basel). 2016; 7(12):genes7120120. 10.3390/genes7120120 PMC5192496 27941642

[64] Haines S, Gautheron S, Nasser W, Renauld-Mongenie G. Identification of Novel Components Influencing Colonization Factor Antigen I Expression in Enterotoxigenic *Escherichia coli*. PLoS ONE. 2015; 10(10):e0141469 10.1371/journal.pone.0141469 26517723 PMC4627747

[65] Haines S, Arnaud-Barbe N, Poncet D, Reverchon S, Wawrzyniak J, Nasser W, et al IscR Regulates Synthesis of Colonization Factor Antigen I Fimbriae in Response to Iron Starvation in Enterotoxigenic *Escherichia coli*. J Bacteriol. 2015; 197(18):2896–907. 10.1128/JB.00214-15 26124243 PMC4542172

[66] Hodson C, Yang J, Hocking DM, Azzopardi K, Chen Q, Holien JK, et al Control of Virulence Gene Expression by the Master Regulator, CfaD, in the Prototypical Enterotoxigenic *Escherichia coli* Strain, H10407. Front Microbiol. 2017; 8:1525 10.3389/fmicb.2017.01525 eCollection;%2017.:1525. 28848532 PMC5554520

[67] Wolf MK, Andrews GP, Tall BD, McConnell MM, Levine MM, Boedeker EC. Characterization of CS4 and CS6 antigenic components of PCF8775, a putative colonization factor complex from enterotoxigenic *Escherichia coli* E8775. Infect Immun. 1989; 57:164–73. 2491834 10.1128/iai.57.1.164-173.1989 PMC313061

[68] Caron J, Maneval DR, Kaper JB, Scott JR. Association of RNs Homologs with Colonization Factor Antigens in Clinical Escherichia-Coli Isolates. Infect Immun. 1990; 58:3442–4. 2205583 10.1128/iai.58.10.3442-3444.1990 PMC313674

[69] Favre D, Ludi S, Stoffel M, Frey J, Horn MP, Dietrich G, et al Expression of enterotoxigenic *Escherichia coli* colonization factors in *Vibrio cholerae*. Vaccine. 2006; 24(20):4354–68. 10.1016/j.vaccine.2006.02.052 16581160

[70] Nicklasson M, Sjoling A, von MA, Qadri F, Svennerholm AM. Expression of colonization factor CS5 of enterotoxigenic *Escherichia coli* (ETEC) is enhanced in vivo and by the bile component Na glycocholate hydrate. PLoS ONE. 2012; 7(4):e35827 10.1371/journal.pone.0035827 22563407 PMC3342736

[71] Nicklasson M, Sjoling A, Lebens M, Tobias J, Janzon A, Brive L, et al Mutations in the periplasmic chaperone leading to loss of surface expression of the colonization factor CS6 in enterotoxigenic *Escherichia coli* (ETEC) clinical isolates. Microb Pathog. 2008; 44(3):246–54. 10.1016/j.micpath.2007.06.009 18037262

