# Supplementary material for: Colonization factors among enterotoxigenic Escherichia coli isolates from children with moderate-to-severe diarrhea and from matched controls in the Global Enteric Multicenter Study (GEMS)
Source: PLoS Negl Trop Dis. 2019 Jan 4;13(1):e0007037. doi: 10.1371/journal.pntd.0007037 (PMC6343939; doi:10.1371/journal.pntd.0007037)
Supplement: S1 Checklist — (DOC) [file pntd.0007037.s001.doc]

STROBE Statement—Checklist of items that should be included in reports of ***case-control studies***

|  | Item No | Recommendation |
| --- | --- | --- |
| **Title and abstract** | 1 | (*a*) Indicate the study’s design with a commonly used term in the title or the abstract  Page 1.  Title: Colonization factors among enterotoxigenic *Escherichia coli* isolates from children with moderate-to-severe diarrhea and from matched controls in the Global Enteric Multicenter Study (GEMS) |
|  |  | (*b*) Provide in the abstract an informative and balanced summary of what was done and what was found  Page 4.  **Abstract.**  **Background.** Enterotoxigenic *Escherichia coli* (ETEC) encoding heat-stable enterotoxin (ST) alone or with heat-labile enterotoxin (LT) cause moderate-to-severe diarrhea (MSD) in developing country children. The Global Enteric Multicenter Study (GEMS) identified ETEC encoding ST as one of the top four enteropathogens. To guide vaccine development, we examined colonization factor (CF) prevalence among ETEC isolates from children age <5 years with MSD and from matched controls in four African and three Asian sites and assessed strength of association of specific CFs with MSD.  **Methodology/Principal Findings.** MSD cases enrolled at healthcare facilities over three years and matched controls had three *E. coli* colonies per child tested by polymerase chain reaction to detect genes encoding LT, ST, major CFs (Colonization Factor Antigen I [CFA/I] or Coli Surface [CS] antigens CS1-CS6) and minor CFs (CS7, CS12, CS13, CS14, CS17, CS18, CS19, CS20, CS21, CS30). ETEC from 806 cases had a single toxin/CF profile in three tested strains per child. Major CFs, detected in 66.0% of LT/ST and ST-only cases, were significantly associated with MSD versus matched controls by conditional logistic regression (p<0.006); major CFs were detected in only 25.0% of LT-only cases and were not associated with MSD. ETEC encoding exclusively CS14, identified among 19.9% of 291 ST-only and 1.5% of 259 LT/ST strains, were associated with MSD (p=0.0011). No other minor CF exhibited a prevalence >5% and exhibited a significant association with MSD.  **Conclusions/Significance.** Major CF-based efficacious ETEC vaccines could prevent ~66% of pediatric MSD cases due to ST-encoding ETEC in developing countries; adding CS14 would extend coverage to ~77%. |
| Introduction | | |
| Background/rationale | 2 | Explain the scientific background and rationale for the investigation being reported  Page 6-8.  Enterotoxigenic *Escherichia coli* (ETEC) cause diarrheal disease in children <5 years of age in developing countries and travelers’ diarrhea among persons from industrialized countries who visit developing countries [1,2]. Human ETEC strains can produce a heat-labile enterotoxin (LT) that resembles cholera toxin and one or more heat-stable enterotoxins (ST) including human ST (STh) or porcine ST (STp). Strains can produce both LT and ST (LT/ST) or be ST-only or LT-only. Most ETEC encode colonization factors (CFs) that allow the pathogen to attach to proximal small intestine enterocytes, the critical site of host-parasite interaction, before expressing enterotoxins that decrease villus tip cell absorption and evoke secretion of electrolytes and water by crypt cells [3].  Three main families of Colonization Factor Antigens (CFAs) are encoded by ETEC that cause diarrhea in humans including CFA/I, CFA/II and CFA/IV [3]. CFA/I is the sole member of the first family. CFA/II strains encode coli surface (CS) antigen 3 (CS3) alone or in combination with CS1 or CS2 [3], while CFA/IV strains encode CS6 alone or in conjunction with CS4 or CS5 [3]. CFA/I, CS1, CS2, CS4 and CS5 are rigid fimbriae ~6-7 nm in diameter, CS3 are thin flexible fibrillae 2-3 nm in diameter [4], and CS6 morphology is nondescript.  ETEC vaccines intending to stimulate anti-CF immunity, with or without accompanying antitoxic immunity, are in clinical development. These include purified fimbriae or tip adhesins [5], inactivated fimbriated ETEC [6], attenuated ETEC expressing CFs [7], bacterial live vectors such as *Shigella* encoding ETEC CFs [8], multiple epitope fusion antigens [9], and ST toxoids [10]. Stimulating intestinal secretory IgA antibodies that bind CFs and prevent ETEC from attaching to human small intestine mucosa is generally considered to be fundamental to a successful ETEC vaccine, although some contend that parenteral vaccine-induced serum IgG antibodies that transude onto intestinal mucosa may also prevent diarrhea in humans caused by bacterial enteropathogens [11]. Most current ETEC vaccines contain a mix of antigens directed against CFA/I, CFA/II and CFA/IV antigens.  Minor putative CFs also exist for which data supporting their role in pathogenesis in humans is less compelling or lacking, although they also mediate attachment to human cells in tissue culture. Possible exceptions are CS17 LT-only strains that evoked diarrhea in challenged volunteers [12] and LT-only isolates encoding CS7 that were incriminated in small cohort studies of pediatric diarrhea [13,14]. While minor CF antigens CS7, CS12, CS14, CS17, CS18, CS19, CS20, CS21 and CS30 have received most attention, others have also been described including CS8, CS10, CS11, CS13, CS15 and CS23 [15-19].  DNA-based high-throughput diagnostics have enabled large epidemiologic studies to quantify the ETEC disease burden among young children in developing countries and to assess the prevalence of various CFs. The Global Enteric Multicenter Study (GEMS) tested for a large number of diarrheal pathogens, including ETEC, among cases of moderate-to-severe diarrhea (MSD) and matched (by age, gender, neighborhood and time of presentation) control children without diarrhea in three age strata (0-11, 12-23 and 24-59 months) at four sites in sub-Saharan Africa and three in South Asia [1,20], the geographic regions where 80% of global diarrheal deaths occur. ST-producing ETEC, i.e., LT/ST and ST-only strains, were significantly incriminated as pathogens and placed ETEC as one of the top four pathogens associated with MSD across all seven sites and age groups [1].  Herein we present the proportion of GEMS ETEC isolates that encode the main CFs found in most ETEC vaccines under development, and the prevalence of multiple other putative attachment factors (CS7, CS12, CS13, CS14, CS17, CS18, CS19, CS20, CS21, CS30) that have been proposed as potential antigens to broaden ETEC vaccine immunoprophylaxis. In addition, based on the GEMS case/control design, we present the strength of association with MSD of ETEC of the different toxin genotypes encoding various CFs. |
| Objectives | 3 | State specific objectives, including any prespecified hypotheses  Page 8.  Herein we present the proportion of GEMS ETEC isolates that encode the main CFs found in most ETEC vaccines under development, and the prevalence of multiple other putative attachment factors (CS7, CS12, CS13, CS14, CS17, CS18, CS19, CS20, CS21, CS30) that have been proposed as potential antigens to broaden ETEC vaccine immunoprophylaxis. In addition, based on the GEMS case/control design, we present the strength of association with MSD of ETEC of the different toxin genotypes encoding various CFs. |
| Methods | | |
| Study design | 4 | Present key elements of study design  Page 8 and 9.  **Study design and population**  The rationale [20], assumptions, clinical, epidemiological and microbiological methods of GEMS [1,21], a three-year case-control study undertaken among children <5 years of age in Gambia (Basse), Mali (Bamako), Mozambique (Manhiça) and Kenya (Siaya County) in sub-Saharan Africa and India (Kolkata), Bangladesh (Mirzapur) and Pakistan (Karachi-Bin Qasim Town) in South Asia, have been described. MSD was defined as an acute episode of diarrhea (>3 loose stools during a 24-hour period) that started within the previous seven days, was separated from another episode by >7 days, and was accompanied by either signs of dehydration (sunken eyes, slow abdominal “skin pinch” recoil or administration of intravenous fluids), dysentery or admission to hospital based on clinical concern over diarrheal disease severity [1,22]. |
| Setting | 5 | Describe the setting, locations, and relevant dates, including periods of recruitment, exposure, follow-up, and data collection  Page 8.  This three-year case-control study was undertaken among children <5 years of age in Gambia (Basse), Mali (Bamako), Mozambique (Manhiça) and Kenya (Siaya County) in sub-Saharan Africa and India (Kolkata), Bangladesh (Mirzapur) and Pakistan (Karachi-Bin Qasim Town) in South Asia.  The details of the seven study sites are described in Kotloff KL et al. Lancet 2013; 382:209-22, Kotloff KL et al. Clin Infect Dis 2012; 55 Suppl4:S232-245, and Levine MM et al. Clin Infect Dis 2012; 55 Suppl4:S215-224. In each site a defined population served by sentinel health care facilities was under demographic surveillance so that births, deaths and migrations in and out were detected by visiting all households 2-3 times annually. |
| Participants | 6 | (*a*) Eligibility criteria, sources and methods of case ascertainment and control selection and rationale for the choice of cases and controls.  Page 8.  The details of the eligibility criteria are described in detail in Kotloff KL et al. Lancet 2013; 382:209-22, Kotloff KL et al. Clin Infect Dis 2012; 55 Suppl4:S232-245 and Levine MM et al. Clin Infect Dis 2012; 55 Suppl4:S215-224.    The GEMS was carried out in three age strata including infants (age 0-11 months), toddlers (12-23 months) and pre-school children (24-59 months of age).  Only children with moderate-to-severe diarrhea (MSD) were eligible. This study was designed to enrol cases of a severity such that if they did not have access to health care (as is common among pediatric populations in under-resourced developing countries) the cases might have been fatal. Thus, GEMS aimed to elucidate the etiology and measure the burden of more severe forms of diarrheal illness in young children.  MSD was defined as an acute episode of diarrhea (>3 loose stools during a 24-hour period) that started within the previous seven days, was separated from another episode by >7 days, and was accompanied by either signs of dehydration (sunken eyes, slow abdominal “skin pinch” recoil or administration of intravenous fluids), dysentery or admission to hospital based on clinical concern over diarrheal disease severity. |
|  |  | (*b*) Matching criteria and the number of controls per case.  Page 8.  As described in detail in the main GEMS references that provide details on the clinical methods (Kotloff KL et al [Lancet 2013; 382:209-22] and Kotloff KL et al [Clin Infect Dis 2012; 55 Suppl4:S232-245], “we aimed to enrol one to three control children without diarrhoea during a home visit, following an algorithm that increased the requisite controls according to the number of patients with moderate-to-severe diarrhoea enrolled in that fortnight. Controls, matched to every individual patient with moderate-to-severe diarrhoea by age (±2 months for patients aged 0–11 months and 12–23 months, and ±4 months for patients aged 24–59 months), sex, and residence (same or nearby village or neighbourhood as the patient with diarrhoea), were randomly selected from the site’s DSS database and enrolled within 14 days of the index case. Potential controls who had diarrhoea in the previous 7 days were ineligible.” |
| Variables | 7 | Clearly define all outcomes, exposures, predictors, potential confounders, and effect modifiers. Give diagnostic criteria, if applicable  Page 9-14 and Tables 1 and 2.  As a component of GEMS, we undertook to examine the prevalence of the different types of major and minor colonization factors among enterotoxigenic *E. coli* (ETEC) isolated from MSD cases and controls enrolled in the study. At the sites, three *E. coli* colonies were tested with specific primers to amplify the genes encoding heat-labile enterotoxin (LT) and heat-stable enterotoxin. The standardized PCR methods used at the seven GEMS site to detect ETEC are described in Panchalingam S. et al [Clin Infect Dis 2012; 55 Suppl4:S294-302].  All positive ETEC isolates were sent to a GEMS Reference Laboratory at the University of Chile in Santiago, Chile, where each isolate was re-tested to define its enterotoxin genotype (using primers for LT, STh and STp) and to amplify genes encoding the major ETEC colonization factors.  Only MSD cases and controls that had a single toxin genotype among all three of their triplet isolates were further analysed by PCR to detect the presence of genes encoding CFA/I, CFA/II family (CS3-only, CS3+CS1 or CS3+CS2) and the CFA/IV family (CS6-only, CS6+CS4 or CS6+CS5), i.e., the major colonization factors. In total, ETEC isolate triplets from 806 of 894 cases (90.2%) had a homogeneous genotype across all three isolates, as did triplets form 711 of 975 controls (72.9%) with confirmed ETEC.  Any case or control that was PCR positive for CFA/I, CFA/II family (CS3-only, CS3+CS1 or CS3+CS2) or CFA/IV family (CS6-only, CS6+CS4 or CS6+CS5) were considered to be positive for a major CF. Isolates that were negative for major CFs were then further tested by PCR to detect the 10 minor CFs that have generated the most interest in the literature as potential antigens to be included in a CF-based ETEC vaccines. These include CS7, CS12, CS13, CS14, CS17, CS18, CS19, CS20, CS21 and CS30. Since one objective was to determine the potential value of adding one or more minor CFs to a CF-based ETEC vaccine, we looked for ETEC isolates from cases that were negative for major CFs and encoded only a single minor CF. We a priori determined that strains expressing only a single minor CF at a prevalence of less than 5% within a toxin genotype would be of little practical interest. |
| Data sources/ measurement | 8* | For each variable of interest, give sources of data and details of methods of assessment (measurement). Describe comparability of assessment methods if there is more than one group  Pages 9-14. Table 1. Figures 1 and 2.  The enterotoxin genotypes and encoding of major and minor CFs were determined by PCR methodology using established primers (Table 1) except for new primers that were designed to detect CS30 and improved primers to detect CS18 with greater specificity. All primer sets are shown in Table 1 and the new primers prepared in this study are described in the laboratory methods and shown in figure 1 and figure 2. |
| Bias | 9 | Describe any efforts to address potential sources of bias  It was necessary to have a strategy to capture cases of MSD in a practical way. The assumption made by the designers of the GEMS-1 project is that cases of diarrheal disease for which healthcare is sought at hospitals or health centers are likely to be more severe than cases that might be detected at the household level by systematic active visitations. Thus, the assumption was that passive surveillance at sentinel ﬁxed healthcare facilities would be enriched for MSD cases. Accordingly, the selection of the most appropriate sentinel healthcare facilities was a critical step in the GEMS-1 design. As described by Kotloff et al [Clin Infect Dis 2012; 55 Suppl4:S232-245], a large baseline random survey (Health Care Utilization and Attitudes Survey [HUAS]) carried out within the linked deﬁned population generated the necessary information to identify the most appropriate facilities. This survey also provided information on healthcare preferences to allow adjustment so that populationwide incidence rates could be generated based on extrapolation of data gathered from sentinel health centers (SHCs).  An age-, sex- and village or neighborhood-matched control selection strategy was selected to limit potential confounding by factors that may not be easily controlled for in statistical analysis, thus ensuring the integrity of the results. This type of study must be meticulously designed both with respect to identiﬁcation of the MSD cases and selection of matched controls.  Besides the matched case/control study, a Health Care Utilization and Attitudes Survey (HUAS), based on random sampling from each site’s demographic surveillance system (DSS), was conducted before the beginning of the GEMS case/control study. Truncated versions of the HUAS, known as HUAS-lite, were conducted several times during the 3 years of case and control enrollment. The HUAS was used to evaluate associations between a variety of demographic factors and characteristics of households (eg, main source of water, main method of disposal of feces) with the presence of diarrhea in the sampled child. The HUAS-lite surveys were used primarily to estimate the proportion of children with MSD who were taken to one of the site’s sentinel health centers and to estimate the 1-week incidence of MSD.  Of the various statistical analyses in GEMS, it is the analysis of etiology that is the most important and demanding. The reason is that one of the driving rationales for initiating the GEMS was to be able, on the basis of the results, to prioritize the allocation of ﬁnancial and other resources toward the implementation of existing interventions (such as vaccines and therapeutics) and to prioritize investments in research aimed at developing new interventions, based on the relative contributions of different pathogens to the overall burden of MSD in young children. From this perspective, one sees clearly the potential utility of the attributable fraction (AF), deﬁned as the proportion of MSD that would be eliminated if the target population were no longer exposed to a speciﬁc risk factor (such as a speciﬁc pathogen). AF allows us to estimate the number of MSD cases at one of our sites that can be attributed to a speciﬁc pathogen, adjusted for other pathogens that might also be present. Thus, we can distinguish between a pathogen that is responsible for a large number of cases and another pathogen that might be associated with MSD but for which the number of attributable cases is considerably smaller. This is crucial in allowing policy makers to set priorities for interventions. Further, it can help us to identify locations where a speciﬁc intervention might make a large impact and other locations where its impact might be relatively minor.  The following is from the primary GEMS report in Lancet (Kotloff K et al 2013):    We assessed associations of moderate-to-severe diarrhoea with potential pathogens using conditional logistic regression with a penalised likelihood approach; taking into account the presence or absence of multiple pathogens as independent variables, we used odds ratios (ORs) and pathogen prevalence among patients with moderateto-severe diarrhoea to calculate adjusted population attributable fractions (AFs)^12^ to estimate pathogenspeciﬁ c disease burden (expressed as number of cases and incidence rate). The adjusted AF is derived from a multiple conditional logistic regression model that includes other pathogens signiﬁcantly associated with moderate-to-severe diarrhoea; thus it is the AF adjusted for presence of other pathogens. Because samples from patients with moderate-to-severe diarrhoea were taken in roughly equal numbers during each fortnight, irrespective of the number of cases of moderate-to-severe diarrhoea appearing at the SHCs, we estimated AF using weights deﬁ ned as the number of eligible cases divided by the number of enrolled cases— ie, as the inverse of the sampling fraction for cases. **We calculated weights separately for cases of MSD with and without dysentery, to avoid any bias from overrepresentation or underrepresentation of cases with dysentery.** We combined data for two or more adjacent fortnights to avoid having periods with either no patients with diarrhoea and dysentery enrolled or no patients with diarrhoea and without dysentery enrolled; typically, this resulted in the combination of data for eight adjacent periods, so that weighting was based on numbers of cases in 16 weeks. To estimate disease burden of moderate-to-severe diarrhoea in the population, we did brief surveys of health-care use serially during the case-control study in concert with each round of the DSS, using random samples of children. We asked the parent or primary caretaker of children enrolled in every survey whether her or his child had a new episode of moderate-to-severe diarrhoea during the preceding 7 days, and, if so, the type of health care sought. After pooling data from serial surveys at each site, and applying sampling weights for surveyed children based on the number of children in each age–sex stratum in the DSS population, we calculated the proportion, designated “r”, of children with moderate-to-severe diarrhoea who were taken to an SHC at each site within 7 days of onset of diarrhoea. For each site and age stratum, we estimated numbers of cases and incidence rates of moderate-to-severe diarrhoea per 100 child-years as follows. We calculated the annual number of cases of moderate-to-severe diarrhoea in the population as the number of eligible cases of moderate-tosevere diarrhoea recorded at SHCs during the 36-month study, divided by 3×r (with “r” deﬁned as above). Division of this result by the median DSS population gave the moderate-to-severe diarrhoea incidence rate. To derive the number of cases and the incidence of moderate-to-severe diarrhoea attributable to a speciﬁc pathogen, the total cases and incidence rates of moderate-to-severe diarrhoea were multiplied by the pathogen’s weighted AF.  The sentences below are from reference 28, Blackwelder WC et al (2012):  The most important limitation regarding statistical analysis of GEMS data is probably in the estimation from HUAS-lite surveys of the proportion of MSD cases taken to an SHC, which we call “r.” This proportion is important in our estimation of total MSD burden, as well as the burden attributable to speciﬁc pathogens (such as ETEC). The HUAS-lite surveys were based on random samples from the DSS population. However, for various reasons (e.g., in Kolkata many MSD cases were taken to private healthcare providers) in most sites and age groups r is smaller than we had hoped it would be (<40% in all except 1 site). Thus, there is the potential for bias in the proportion of cases enrolled with a speciﬁc manifestation of MSD or in which a speciﬁc pathogen was isolated. |
| Study size | 10 | Explain how the study size was arrived at.  Reference 28  As summarized by Blackwelder et al [Clin Infect Dis 2012; 55 Suppl4:S246-253], the three age strata studied in GEMS represent age groups in which MSD and its clinical presentations (e.g., dysentery) are observed with different frequencies and when the etiologies are known to be somewhat different. For example, based on pre-GEMS data, certain etiologies are relatively more important in infants, while others are more common in toddlers or preschool children with MSD. Most of the analyses were intended to be done within these age strata. Calculation of statistical power was based on comparing 2 independent proportions; for a moderate degree of correlation between presence of a pathogen in a case and in its matched control, the power for a given sample size will be higher for a test designed for matched data. The planned sample size at each site was 600 analyzable case/control pairs. This sample size should be sufﬁcient, for example, for a test at the 2-sided 5% signiﬁcance level to have 80% power to ﬁnd a signiﬁcant difference between proportions of cases and controls for which a speciﬁc pathogen is isolated, if the respective true proportions are 5.8% and 2.5%.  References 1 & 22.  As described in detail in the main GEMS references that provide details on the clinical and epidemiological methods (Kotloff KL et al [Lancet 2013; 382:209-22] and Kotloff KL et al. [Clin Infect Dis 2012; 55 Suppl4:S232-245], “Although all cases meeting the deﬁnition of moderate-to-severe diarrhoea were documented, each site restricted enrolment to about the ﬁrst nine eligible cases per age stratum per fortnight to maintain a manageable work ﬂow throughout the study. We attempted to enroll 600 analyzable case-control pairs per age stratum per site in 36 months, which would provide 80% power (two-sided test, 5% signiﬁcance level) to ﬁnd a signiﬁcant diﬀerence for a site-stratum-speciﬁc comparison of the proportion of cases and controls in whom a speciﬁc enteropathogen is identiﬁed, if a pathogen is identiﬁed in 5.8% of cases and 2.5% of controls. |
| Quantitative variables | 11 | Explain how quantitative variables were handled in the analyses. If applicable, describe which groupings were chosen and why.  Page 14.  Prevalences of ETEC CFs were expressed as percentages in a stratified analysis by ETEC toxin profile, site and region. |
| Statistical methods | 12 | (*a*) Describe all statistical methods, including those used to control for confounding.  Reference 28 and reference 1.  The statistical methods used in the main GEMS case/control study are described in detail in reference 28: Blackwelder et al. Statistical Methods in the Global Enteric Multicenter Study (GEMS). Clin Infect Dis. 2012; 55 Suppl 4:S246-53. doi: 10.1093/cid/cis788.:S246-S253 and in reference 1 (Kotloff KL et al. Lancet 2013).  Page 14 & 15.  The strength of association between ETEC toxin and CF genotypes and MSD was examined using conditional logistic regression models in which the outcome was case-control status (MSD) and the independent variable (covariate) was whether the child’s ETEC had the specific CFA (no/yes) (reference 28), while applying Firth’s penalized likelihood approach. Matched odds ratios (ORs) and corresponding 95% confidence intervals (CIs) were obtained from these models. Pooled and site-specific analyses were conducted. Heterogeneity in ORs across sites was examined using Chi square test for heterogeneity. A p <0·05 was considered significant. Data were analyzed using SPSS version 23 (IBM, Inc., Armonk, NY) and SAS statistical software version 9·4 (SAS Institute Inc. Cay, NC, USA).  Page 26. Lines 350-352.  We did not use a Bonferroni adjustment for the ten individual conditional logistic regression analyses of the association of individual minor CFs with MSD, as in each instance an individual hypothesis was being tested [31-34]. |
|  |  | (*b*) Describe any methods used to examine subgroups and interactions  See reference 1, Kotloff KL et al. Lancet 2013; 382:209-22. |
|  |  | (*c*) Explain how missing data were addressed  There was no imputation of missing data. |
|  |  | (*d*) If applicable, explain how matching of cases and controls was addressed  References 1 and 22.  As described in detail in the main GEMS references that provide details on the clinical methods (Kotloff KL et al (Lancet 2013; 382:209-22) and Kotloff KL et al. Clin Infect Dis 2012; 55 Suppl4:S232-245, “we aimed to enroll one to three control children without diarrhoea during a home visit, following an algorithm that increased the requisite controls according to the number of patients with moderate-to-severe diarrhoea enrolled in that fortnight. Controls, matched to every individual patient with moderate-to-severe diarrhoea by age (±2 months for patients aged 0–11 months and 12–23 months, and ±4 months for patients aged 24–59 months), sex, and residence (same or nearby village or neighbourhood as the patient with diarrhoea), were randomly selected from the site’s DSS database and enrolled within 14 days of the index case. Potential controls who had diarrhoea in the previous 7 days were ineligible.” |
|  |  | (*e*) Describe any sensitivity analyses  In the main GEMS study we attempted to enrol 600 analyzable case-control pairs per age stratum per site in 36 months, which would provide 80% power (two-sided test, 5% signiﬁcance level) to ﬁnd a signiﬁcant diﬀerence for a site-stratum-speciﬁc comparison of the proportion of cases and controls in whom a speciﬁc enteropathogen is identiﬁed, if a pathogen is identiﬁed in 5.8% of cases and 2.5% of controls. |
| Results | | |
| Participants | 13* | (a) Report numbers of individuals at each stage of study—eg numbers potentially eligible, examined for eligibility, confirmed eligible, included in the study, completing follow-up, and analysed  Reference 1 (Kotloff KL et al. Lancet 2013; 382:209-22).  Within each age stratum of the overall GEMS study, the numbers of children who were potentially eligible (i.e., visiting the sentinel health centers with a complaint of diarrheal illness), examined for eligibility, confirmed as eligible, enrolled into the study, completed follow-up and analysed are contained within Figures 1, 2 and 3 of reference 1 (Kotloff et al. Lancet 2013; 382:209-22). |
|  |  | (b) Give reasons for non-participation at each stage  See Figures 1, 2 and 3 of reference 1 (Kotloff et al. Lancet 2013; 382:209-22) in the submitted manuscript. |
|  |  | (c) Consider use of a flow diagram  Figures 1, 2 and 3 of reference 1 (Kotloff et al. Lancet 2013; 382:209-22) in the submitted manuscript are the flow diagrams. |
| Descriptive data | 14* | (a) Give characteristics of study participants (eg demographic, clinical, social) and information on exposures and potential confounders  Reference 1.  Characteristics of the GEMS study case and control participants are described in Table 1 of reference 1 (Kotloff et al. Lancet 2013; 382:209-22) cited in the submitted manuscript. |
|  |  | (b) Indicate number of participants with missing data for each variable of interest  Table 5 and Table 6.  A step-wide approach was used to testing ETEC strains from cases that lacked major CFs to identify isolates that encode just a single minor CF. The prevalence of such strains was determined (Table 5). In this step-wise testing there were a few *E. coli* strains that could not be revived from frozen stock to culture and extract additional DNA for further testing. These few missing strains are shown as footnotes in Table 5. |
| Outcome data | 15* | Report numbers in each exposure category, or summary measures of exposure  Table 2 (page 16), Table 3 (pages 17-18) and Table 4 (20-21)  Tables 2-4 show the toxin genotypes and the prevalences of CFs among ETEC of different toxin genotypes from cases and controls, by country, by continent (Africa and South Asia) and all sites pooled. |
| Main results | 16 | (*a*) Give unadjusted estimates and, if applicable, confounder-adjusted estimates and their precision (eg, 95% confidence interval). Make clear which confounders were adjusted for and why they were included  Table 5 (page 23).  Among the 550 ETEC isolates encoding ST (including 291 ST-only and 259 LT/ST), 363 (66%) encoded a major CF. In contrast, only 25% of 256 LT-only isolates encoded a major CF and those were mainly CS6.  Among the ST-only isolates from cases, 58 encoded CS14 in the absence of encoding any other CF. This is a very important finding as it identifies a CF antigen in 58 of the 104 ST-only isolates (55.6%) that were negative for a major CF.  Table 6. Page 25  Conditional logistic regression analyses were run for each major CF or CF family and for each minor CF (Table 6). Only CS14 had a prevalence above 5% among ETEC case isolates lacking a major CF (Table 5) and was significantly associated with MSD (p=0.0011) (Table 6). The other minor CFs were also analysed after the data were pooled (but excluding CS14) to determine if collectively, with increased statistical power, the collection of those minor CF strains appeared to be associated with MSD even if individual genotypes were not. The pooled results did not show a significant association with MSD (Table 6). 95% Confidence Intervals are provided around the pooled matched odds ratios shown in Table 6. |
|  |  | (*b*) Report category boundaries when continuous variables were categorized |
|  |  | (*c*) If relevant, consider translating estimates of relative risk into absolute risk for a meaningful time period  This is not applicable. |

| Other analyses | 17 | Report other analyses done—eg analyses of subgroups and interactions, and sensitivity analyses  This is not applicable. |
| --- | --- | --- |
| Discussion | | |
| Key results | 18 | Summarise key results with reference to study objectives  Pages 28-32  Four cardinal findings emerged:  i) GEMS results confirm that ETEC vaccines based on stimulating immune responses to the major CFs (CFA/I and CS1-6), if efficacious in blocking CF-mediated attachment ot enterocytes, could prevent diarrhea caused by ~66% of the ST-only and LT/ST strains incriminated as pathogens (Table 5). The fact that ETEC encoding these CFs were observed in a very large study involving multiple representative sites in sub-Saharan Africa and South Asia validates that ETEC vaccine strategy for the geographic regions where 80% of young child diarrheal deaths occur worldwide.  ii) The second cardinal observation derives from determining the proportion of strains in each toxin genotype that lacked a major CF but that exclusively expressed one minor CF, including either CS7, CS12, CS13, CS14, CS17, CS18, CS19, CS20, CS21 or CS30. Collectively these minor CFs raised the percent of ST-only cases having a recognized CF from 64.3% (187/291) to 86.3% (251/291) and raised the percent of LT/ST cases having a recognized CF from 68.0% (176/259) to 79.9% (207/259) (**Table 5**); the percent of MSD cases with LT-only ETEC having a recognized CF similarly rose from 25.0% (64/256) to 54.7% (140/256).  iii) The third cardinal observation was assessment of the strength of association between MSD and ETEC encoding the various major and minor CFs within the different toxin genotypes.  iv) The fourth key observation is that inclusion of CS14 expands the breadth of vaccine coverage against ST-only pathogens, raising it from 64.3% to 84.2%. |
| Limitations | 19 | Discuss limitations of the study, taking into account sources of potential bias or imprecision.  Page 33.  One theoretical limitation of our study is that the PCR primers designed to detect ST at the field sites were optimized for STh; thus some STp-only isolates may have been missed. LT-STp strains from cases were not under-estimated in GEMS because all LT-only strains were re-tested with PCRs individually optimized for STp and STh in the Chilean Reference Laboratory and upon re-testing only a limited number of LT-only isolates were found to be LT/STp. We believe that few cases and controls with STp-only were missed. A GEMS follow-on study detected STp and STh in genomic DNA extracted from whole stool specimens of a subset of 5304 case/matched control pairs using a TaqMan Card-based quantitative real-time PCR (qPCR) methodology and documented that the ST burden was overwhelmingly attributed to STh [35]. Optimized detection of STp by qPCR increased the overall ETEC disease burden estimate by only 15% versus what was recorded using the gel-based PCR methodology at the field sites [35]. |
| Interpretation | 20 | Give a cautious overall interpretation of results considering objectives, limitations, multiplicity of analyses, results from similar studies, and other relevant evidence  Page 4, page 28 and Page 33.  Major CF-based efficacious ETEC vaccines could prevent ~66% of pediatric MSD cases due to ST-encoding ETEC in developing countries; adding CS14 would extend coverage to ~77%. The major CFs and CS14 are significantly associated with MSD. |
| Generalisability | 21 | Discuss the generalisability (external validity) of the study results  Page 28 (line 398) and Page 29 (lines 399-402).  The fact that the prevalences of case ETEC encoding the major and minor CFs and the significant associations of major CFs and of minor CF CS14 with MSD were observed in a very large study involving multiple representative sites in sub-Saharan Africa and South Asia validates a CF-based ETEC vaccine strategy for the geographic regions where 80% of young child diarrheal deaths occur worldwide. |
| Other information | | |
| Funding | 22 | Give the source of funding and the role of the funders for the present study and, if applicable, for the original study on which the present article is based  Both the original GEMS case/control study report and the current manuscript that describes the colonization factors of ETEC strains form the GEMS study were funded by grants #38874 and #38874.1 from the Bill & Melinda Gates Foundation. |

*Give information separately for cases and controls.

**Note:** An Explanation and Elaboration article discusses each checklist item and gives methodological background and published examples of transparent reporting. The STROBE checklist is best used in conjunction with this article (freely available on the Web sites of PLoS Medicine at http://www.plosmedicine.org/, Annals of Internal Medicine at http://www.annals.org/, and Epidemiology at http://www.epidem.com/). Information on the STROBE Initiative is available at http://www.strobe-statement.org.
